# Mechanistic Non-Response After Psychotherapy for Anxiety Disorders: A Maintenance-Mechanism-Based Clinical Taxonomy

**DOI:** 10.3390/jcm15114223

**Published:** 2026-05-29

**Authors:** Dawid Sasin, Bernard Rybczynski, Bartosz W. Maj, Joanna Chwaszcz, Michal Pruc, Iwona Niewiadomska, Lukasz Szarpak

**Affiliations:** 1 6th Department of Psychiatry, Mazovian Specialist Health Centre in Pruszkow, 05-802 Pruszkow, Poland; 2 Department of Psychiatry, Health Center of Tomaszow Mazowiecki, 97-200 Tomaszow Mazowiecki, Poland; 3 Institute of Psychology, The John Paul II Catholic University of Lublin, 20-950 Lublin, Poland; 4 Institute of Medical Science, The John Paul II Catholic University of Lublin, 20-708 Lublin, Poland

**Keywords:** anxiety disorders, attachment, avoidance, cognitive-behavioral therapy, exposure therapy, inhibitory learning, neurobiology, precision psychiatry, psychotherapy, treatment matching, treatment resistance

## Abstract

Anxiety disorders are disabling and treated with cognitive-behavioral or exposure-based psychotherapy. However, many patients remain symptomatic, fail to remit, relapse, or discontinue treatment. This narrative review examined whether psychotherapy non-response, defined here as persistent clinically significant anxiety symptoms, avoidance, or functional impairment after an apparently adequate psychotherapy trial, may reflect mismatch between therapeutic mechanisms and the dominant processes maintaining anxiety, and aimed to develop a usable taxonomy of mechanistic non-response. This structured narrative review followed SANRA principles. PubMed/MEDLINE, Scopus, PsycINFO, Web of Science, and the Cochrane Library were searched for peer-reviewed literature published from 1 January 2000 to 30 April 2026, including selected earlier landmark studies. Clinical, experimental, neurobiological, psychophysiological, process, and theoretical evidence were synthesized narratively. Psychotherapy mechanisms were organized around inhibitory learning, cognitive reappraisal, attentional modulation, emotion regulation, avoidance reversal, and interpersonal learning. Anxiety maintenance was multilevel, involving threat neurocircuitry, stress-related learning conditions, intolerance of uncertainty, attentional threat capture, safety behaviors, avoidance reinforcement, developmental adversity, and attachment insecurity. Non-response was framed as mismatch between the dominant maintaining process and the therapeutic mechanism expected to modify it. Six failure modes were identified: impaired inhibitory learning, cognitive rigidity/intolerance of uncertainty, stress-related learning impairment, attentional dysregulation, attachment-related barriers, and chronic avoidance dominance. Psychotherapy non-response in adult anxiety disorders should prompt mechanistic reformulation rather than repetition of the same intervention or labeling as treatment resistance. The taxonomy links recognizable failure signatures to mechanism-matched adaptations: redesigned exposure, uncertainty-focused work, attentional interventions, sequencing when arousal or sleep impairs learning, relational repair, and reduction in avoidance contingencies. The narrative review provides a concise clinical taxonomy and practical mechanism-matched adaptations to guide reformulation and treatment redesign after psychotherapy non-response in routine care. The taxonomy supports mechanism-matched reformulation after psychotherapy non-response and requires prospective validation.

## 1. Introduction

Anxiety disorders occupy a central position in clinical psychiatry because they combine high prevalence, early onset, chronicity, functional impairment, and frequent comorbidity [1,2,3]. In DSM-5-TR and ICD-11, anxiety and fear-related disorders include generalized anxiety disorder, panic disorder, agoraphobia, social anxiety disorder, specific phobia, separation anxiety disorder, and selective mutism, while obsessive–compulsive disorder and post-traumatic stress disorder are classified separately despite overlapping fear-learning and avoidance mechanisms. Epidemiological and global burden studies consistently identify anxiety disorders as among the most common mental disorders and as major contributors to years lived with disability [1,3,4]. Their clinical significance extends well beyond subjective distress. Anxiety disorders are associated with educational and occupational impairment, increased healthcare utilization, elevated risk of depressive disorders and substance use, and substantial reductions in quality of life [3].

Psychotherapy is one of the strongest therapeutic traditions in the treatment of anxiety disorders. Cognitive-behavioral therapy (CBT), including exposure-based variants, has repeatedly demonstrated efficacy across anxiety disorders in randomized controlled trials and meta-analyses [3,5,6]. Disorder-specific protocols for panic disorder, social anxiety disorder, generalized anxiety disorder, agoraphobia, and specific phobia are supported by robust evidence [3]. Acceptance- and mindfulness-based interventions have also shown clinically meaningful effects, although the strength and disorder-specificity of evidence vary [3,7]. Psychodynamic therapies have a smaller but clinically relevant evidence base, particularly for social anxiety disorder and generalized anxiety presentations, and may be useful when anxiety is embedded in enduring interpersonal and affective patterns [3]. Contemporary guidelines generally endorse psychological treatments, especially CBT and exposure-based interventions, as first-line or core interventions for many anxiety disorders [3,7,8].

The paradox is that effective psychotherapies often leave many patients insufficiently improved. Across anxiety disorders, meta-analytic estimates suggest that approximately half of patients achieve response or remission after CBT, depending on definitions, populations, outcome measures, and follow-up periods [5,9]. Non-response rates of approximately 30–50% are therefore not marginal anomalies but routine clinical facts [9,10,11]. Some patients attend diligently, complete homework, understand the cognitive model, and still fail to improve. Others improve during treatment but relapse when stress, context, or interpersonal demands change. Some patients appear to benefit cognitively but remain physiologically hypervigilant; others tolerate exposure in session but continue to avoid in daily life. These patterns are familiar to clinicians and are insufficiently explained by broad labels such as non-adherence or resistance [9].

The term treatment resistance has pragmatic value when it denotes persistent clinically significant symptoms after an adequate trial of evidence-based treatment [10,11]. However, in psychotherapy, it is conceptually more complicated than in pharmacotherapy. Psychotherapy is not a uniform dose delivered to a passive biological substrate. It is an interpersonal and learning-based intervention whose effectiveness depends on whether therapeutic procedures activate and modify the processes maintaining the disorder [12,13,14]. A patient may appear resistant because exposure was too narrow to produce inhibitory learning, because cognitive restructuring was mismatched to intolerance of uncertainty, because high stress impaired extinction retrieval, because attentional capture by interoceptive cues was not addressed, or because attachment insecurity rendered the therapeutic relationship itself threatening [12,13,14]. In such cases, failure is not located solely in the patient. It lies in the fit between pathogenesis and intervention [15,16].

The central argument of this review is that psychotherapy failure in anxiety disorders may often reflect a mismatch between therapeutic mechanisms and the neurobiological, cognitive, behavioral, or interpersonal processes maintaining anxiety [12,16]. This does not mean that patient factors are irrelevant. Motivation, adherence, comorbidity, therapist competence, access, culture, and treatment fidelity all remain clinically important [11,17]. Nor does it imply that every treatment failure can be reduced to a single mechanism. Anxiety disorders are heterogeneous, and mechanisms frequently interact [8,18]. The more useful clinical question after an adequate evidence-based psychotherapy has failed is therefore not simply whether the patient is resistant, but which pathogenic process may not have been engaged, modified, or consolidated by the treatment [12,14,19].

The argument developed here is not simply that anxiety treatment should be individualized. That position is already well established. Clinicians routinely recognize that two patients with the same diagnosis may require different therapeutic emphases, and contemporary approaches such as process-based therapy, precision psychotherapy, moderator and mediator research, and inhibitory-learning models of exposure have all contributed substantially to this view [12,14,16,19,20].

The present review addresses a narrower and more clinically specific problem: what should be inferred when a psychotherapy that appears adequate in dose, structure, diagnostic relevance, and delivery of active therapeutic procedures does not produce expected clinical or functional improvement [9,11]. In routine practice, such cases are common. A patient may attend regularly, understand the treatment rationale, complete some assignments, and still remain substantially impaired. Another may improve in the consulting room but relapse rapidly when exposed to stress, interpersonal conflict, bodily arousal, or uncertainty [8,21,22]. These situations are often described as treatment resistance, partial response, poor adherence, or comorbidity. Each description may be partly accurate, but none is sufficiently explanatory [9,11].

This review treats non-response as clinically informative rather than merely as an unfavorable endpoint [12,14,19]. The pattern of failure may indicate which pathogenic process has not been adequately addressed by treatment. Exposure may have been delivered, but the patient may not have acquired retrievable inhibitory learning [13,14]. Cognitive restructuring may have been performed, but the central problem may have been intolerance of uncertainty rather than distorted probability estimation [23,24]. A patient may have completed social exposures while remaining absorbed in self-monitoring. Another may have been too sleep-deprived, hyperaroused, or dissociative to consolidate new learning [21,22,25,26]. In some patients, the therapeutic relationship itself may activate shame, mistrust, or attachment threat, so that technically correct interventions are experienced as criticism, coercion, or abandonment [15,27].

The framework proposed in this review therefore differs from adjacent models in its clinical point of entry. Process-based therapy asks which process should be targeted [16,19,20]. Precision psychotherapy asks which treatment is likely to work for which patient. Mediator research asks through which mechanisms symptom change occurs. Inhibitory-learning models ask how exposure can be optimized [12,14,28]. The present review asks a more specific question: when psychotherapy has failed, what does the failure reveal about the mechanism maintaining the disorder? The distinction between the present framework and adjacent approaches is summarized in Table 1.

This review develops the argument in three steps. First, it describes psychotherapy in terms of mechanisms of action rather than brand names or schools [12,13,14,19]. Second, it summarizes anxiety pathogenesis as a multilevel system involving threat neurocircuitry, stress physiology, cognitive biases, avoidance learning, and interpersonal-developmental processes [8,18,29,30]. Third, it proposes a maintenance-mechanism-based taxonomy of mechanistic non-response and an integrative treatment-matching model [16,19,20]. The aim is not to replace diagnostic frameworks or manualized therapies, but to refine clinical formulation so that non-response becomes a signal for mechanistic reassessment rather than an implicit judgment of the patient [9,11,19].

In clinical practice, the relevant question after non-response is rarely whether the patient is resistant in a global sense. The more useful question is what the treatment did not reach [12,14,19]. Did exposure test the feared expectancy [12,14]? Did cognitive work reduce reassurance seeking or merely refine it [23,31]? Was the patient able to learn under the prevailing level of arousal and sleep disruption [21,22,25,26]? Did the therapeutic relationship feel safe enough for the patient to engage honestly [32]? Did avoidance remain more reinforcing than approach? These questions form the basis of the taxonomy proposed in this review.

## 2. Materials and Methods

This article was designed as a structured narrative review and conceptual synthesis. A narrative approach was selected because the objective was not to estimate a pooled treatment effect, but to integrate evidence from psychotherapy outcome research, experimental psychopathology, translational neuroscience, psychophysiology, neuroimaging, and clinical theory in order to explain clinically distinct patterns of psychotherapy non-response in anxiety disorders. The review was conducted in accordance with the principles of the Scale for the Assessment of Narrative Review Articles (SANRA), with an emphasis on a clearly defined rationale, explicit aims, transparent literature search, appropriate referencing, scientific reasoning, and balanced presentation of evidence [33]. A criterion-by-criterion SANRA self-check is provided in Supplementary Table S1 as a transparency aid; it should not be interpreted as an independent external quality rating.

The review question was formulated as follows: when an apparently adequate evidence-based psychotherapy for an adult anxiety disorder does not produce the expected clinical improvement, which pathogenic processes and disrupted therapeutic mechanisms may explain non-response? The review focused on adult anxiety disorders, including generalized anxiety disorder, panic disorder, agoraphobia, social anxiety disorder, specific phobia, separation anxiety disorder, and clinically relevant anxiety-related presentations. Obsessive–compulsive disorder and post-traumatic stress disorder were not treated as primary diagnostic targets, but selected studies were included when they addressed mechanisms directly relevant to anxiety treatment failure, such as fear extinction, inhibitory learning, avoidance, intolerance of uncertainty, attentional threat processing, emotion regulation, or attachment-related threat. Borderline anxiety-OCD or anxiety-PTSD presentations were handled according to the dominant clinical formulation and the purpose of evidence extraction: studies were retained only when the mechanism under discussion, such as intolerance of uncertainty, metacognitive worry, reassurance seeking, avoidance, attentional threat processing, or fear-extinction processes, was directly relevant to adult anxiety-disorder non-response, whereas studies primarily addressing OCD- or PTSD-specific treatment response were not used to support disorder-specific conclusions for anxiety disorders.

### 2.1. Operational Definitions

Terminology was standardized as follows. Psychotherapy non-response is used as the primary term for the clinical outcome of persistent clinically significant anxiety symptoms, avoidance, distress, or functional impairment after an apparently adequate psychotherapy trial. Mechanistic non-response refers more specifically to the proposed formulation in which non-response is interpreted as possible failure to engage, modify, consolidate, or generalize the dominant maintaining mechanism. Treatment resistance is reserved for the broader clinical and research literature on persistent symptoms after adequate evidence-based treatment and is not used interchangeably with mechanistic non-response.

For the purpose of this review, apparently adequate psychotherapy refers to a completed or substantially completed course of evidence-based psychological treatment that was reasonably matched to the presenting anxiety disorder or anxiety-related formulation and that included the active therapeutic procedures expected to modify the maintaining mechanism [8,10,34]. Adequacy was not defined solely by the number of sessions, because psychotherapy dose varies across disorders, treatment models, and service settings [8,34]. Instead, an intervention was considered apparently adequate when the following minimal conditions were met: the primary anxiety diagnosis or formulation was clinically plausible; the treatment modality had an evidence base for the target presentation; the patient received a sufficient opportunity to engage with the treatment’s active components; therapist competence, treatment structure, or protocol fidelity was not clearly deficient; and major alternative explanations for non-improvement, such as gross diagnostic error, minimal attendance, absence of active therapeutic procedures, severe uncontrolled comorbidity, acute environmental threat, or purely supportive contact misclassified as psychotherapy, were not the dominant explanation for outcome [8,10,11,34,35]. For clinical use, “apparently adequate” psychotherapy was operationalized by a brief adequacy check: plausible anxiety diagnosis or formulation, evidence-based modality, completion or substantial completion of treatment dose, delivery of the relevant active procedures, assessment of between-session implementation and safety behaviors, no clear therapist or fidelity failure, and no dominant alternative explanation such as minimal attendance, gross diagnostic error, severe uncontrolled comorbidity, acute environmental threat, or supportive contact misclassified as psychotherapy.

The qualifier is important. In routine clinical practice and in many psychotherapy studies, information about therapist competence, fidelity, between-session implementation, safety behaviors, and mechanism engagement is often incomplete [8,36,37]. Therefore, this framework does not assume that all reported psychotherapy trials were technically optimal. Rather, it applies to cases in which there is sufficient reason to believe that an evidence-based intervention was delivered with enough dose and structure for therapeutic learning to be expected [8,34].

Non-response was defined as the persistence of clinically significant anxiety symptoms, avoidance, safety behaviors, distress, or functional impairment after an apparently adequate psychotherapy trial, without clinically meaningful improvement on validated symptom measures, clinician-rated global improvement, or clearly observable functional recovery [8,10,34,38,39]. In research contexts, non-response commonly corresponds to failure to meet predefined response or remission criteria, such as insufficient symptom reduction or continued diagnostic-level impairment [10,34,35]. In clinical contexts, the term also includes cases in which symptom scores improve modestly but the maintaining mechanism remains substantially unchanged, for example when the patient reports less distress while continuing to rely on avoidance, reassurance seeking, safety signals, or restricted functioning [8,34,36,39].

This review distinguishes non-response from related but different outcomes. Partial response refers to meaningful but incomplete improvement with persistent residual symptoms, avoidance, or functional impairment [34,40]. Relapse refers to the return of clinically significant anxiety after an initial response or remission [6,34,40,41]. Dropout refers to premature discontinuation before the treatment’s active mechanism has had a reasonable opportunity to operate [34]. These outcomes may overlap clinically, but they have different implications for mechanistic formulation [34,40,41]. The taxonomy proposed here is most applicable to non-response and partial response after an apparently adequate intervention, and less applicable when treatment was too brief, non-specific, poorly matched at the diagnostic level, or discontinued before active therapeutic work occurred [10,11,34].

### 2.2. Information Sources and Search Strategy

A structured literature search was conducted in PubMed/MEDLINE, Scopus, PsycINFO, Web of Science, and the Cochrane Library. The primary search covered publications from 1 January 2000 to 30 April 2026. Earlier landmark papers were included selectively through backward citation searching when they were foundational for the conceptual framework, particularly in relation to emotional processing theory, fear extinction, inhibitory learning, avoidance, and cognitive models of anxiety.

The search strategy combined terms from four domains: anxiety disorders, psychotherapy, non-response or treatment outcome, and candidate mechanisms of treatment failure. Search terms included combinations of the following: anxiety disorder, generalized anxiety disorder, panic disorder, agoraphobia, social anxiety disorder, specific phobia, separation anxiety, cognitive behavioral therapy, CBT, exposure therapy, psychotherapy, psychodynamic therapy, acceptance and commitment therapy, mindfulness-based therapy, treatment response, non-response, remission, relapse, dropout, treatment resistance, fear extinction, inhibitory learning, expectancy violation, safety behaviors, avoidance, intolerance of uncertainty, worry, cognitive reappraisal, threat appraisal, attentional bias, self-focused attention, interoceptive monitoring, emotion regulation, attachment, mentalization, trauma, early adversity, stress, HPA axis, cortisol, amygdala, insula, prefrontal cortex, neuroimaging predictors, biomarkers, moderators, mediators, and precision psychotherapy.

A representative PubMed search string was: (“anxiety disorder*” OR “generalized anxiety disorder” OR “panic disorder” OR “agoraphobia” OR “social anxiety disorder” OR “specific phobia” OR “separation anxiety”) AND (“psychotherap*” OR “cognitive behavioral therapy” OR “CBT” OR “exposure therapy” OR “acceptance and commitment therapy” OR “mindfulness” OR “psychodynamic”) AND (“treatment response” OR “nonresponse” OR “non-response” OR “remission” OR “relapse” OR “dropout” OR “treatment resistance” OR “treatment failure”) AND (“fear extinction” OR “inhibitory learning” OR “expectancy violation” OR “safety behavior*” OR “avoidance” OR “intolerance of uncertainty” OR “worry” OR “cognitive reappraisal” OR “attentional bias” OR “self-focused attention” OR “interoception” OR “emotion regulation” OR “attachment” OR “mentalization” OR “stress” OR “cortisol” OR “HPA axis” OR “amygdala” OR “insula” OR “prefrontal cortex” OR “biomarker*” OR “moderator*” OR “mediator*”).

Search strings were adapted for each database according to its indexing terms and syntax. Reference lists of included systematic reviews, meta-analyses, major theoretical papers, and clinical guidelines were also hand-searched to identify additional relevant studies. The full database-specific search strategies, search dates, pre-deduplication record counts, and mapping of search strings to candidate mechanism domains are provided in Supplementary Table S2.

### 2.3. Eligibility Criteria

Eligible sources included systematic reviews, meta-analyses, randomized controlled trials, longitudinal cohort studies, experimental psychopathology studies, psychotherapy process studies, neuroimaging studies, psychophysiological studies, and major theoretical or conceptual papers with direct relevance to mechanisms of anxiety treatment response or non-response. Clinical guidelines and diagnostic manuals were used for contextual framing but were not treated as primary evidence for mechanisms.

Studies were included when they met at least one of the following criteria:

- Examined psychotherapy response, non-response, remission, relapse, dropout, or treatment resistance in adult anxiety disorders;
- Investigated a mechanism plausibly involved in psychotherapy outcome, including fear extinction, inhibitory learning, avoidance, intolerance of uncertainty, attentional threat processing, cognitive reappraisal, stress physiology, emotion regulation, attachment, or interpersonal learning;
- Clarified how a therapeutic mechanism may fail to engage a pathogenic process maintaining anxiety;
- Provided evidence relevant to treatment adaptation, sequencing, augmentation, or mechanism-based case formulation.

Studies were excluded when they were not peer-reviewed, were not available in English, focused exclusively on pediatric populations without relevance to adult anxiety mechanisms, addressed non-anxiety conditions without a clear mechanistic link to anxiety treatment, lacked sufficient methodological detail, or discussed psychotherapy outcomes without reference to clinical or mechanistic relevance. Case reports were generally excluded unless they were used only to illustrate rare or clinically distinctive phenomena and were not used as primary evidence.

### 2.4. Study Selection

Titles, abstracts, and full texts were screened for relevance to the review question. Records were considered potentially relevant when they addressed psychotherapy outcome, treatment response or non-response, anxiety maintenance mechanisms, or clinically relevant processes such as fear extinction, inhibitory learning, avoidance, intolerance of uncertainty, attentional threat processing, cognitive reappraisal, stress physiology, emotion regulation, attachment, or interpersonal learning.

Because this was a structured narrative review and conceptual synthesis rather than a systematic review or meta-analysis, study selection was guided by clinical and mechanistic relevance rather than exhaustive quantitative aggregation. Priority was given to sources that clarified how a therapeutic mechanism may fail to engage a maintaining process, identified a recognizable clinical pattern of non-response, or informed treatment adaptation, sequencing, augmentation, or mechanism-based case formulation.

The final reference set was reviewed for conceptual coverage across the main domains of the article: psychotherapy mechanisms, multilevel anxiety maintenance, mechanistic failure modes, treatment matching, predictors or biomarkers of response, and clinical implications. Sources were included when they made a substantive contribution to one or more of these domains.

### 2.5. Data Extraction and Evidence Mapping

For each included source, the following information was extracted where applicable: author, year, study type, population, anxiety disorder or presentation, psychotherapy modality, definition of response or non-response, follow-up duration, mechanism assessed, main findings, limitations, and relevance to treatment failure. For experimental and translational studies, extraction focused on the mechanism examined and its clinical relevance to psychotherapy. For systematic reviews and meta-analyses, extraction focused on the consistency, strength, and limitations of the evidence base.

Extracted evidence was mapped onto six candidate failure modes: impaired inhibitory learning, cognitive rigidity, stress-related learning impairment, attentional dysregulation, attachment-related barriers, and chronic avoidance dominance. These categories were refined iteratively during synthesis. A failure mode was retained when it met three criteria: it was supported by a plausible pathogenic process, it corresponded to a disrupted therapeutic mechanism, and it had a recognizable clinical signature with a potential corrective treatment implication.

### 2.6. Evidence Prioritization and Critical Appraisal

Given the heterogeneity of the evidence base, formal meta-analysis and pooled effect estimation were not performed. Evidence was prioritized according to methodological strength and relevance to the review question. Highest priority was given to systematic reviews, meta-analyses, randomized controlled trials, well-designed longitudinal studies, experimental studies with direct relevance to therapeutic learning, and replicated psychotherapy process findings. Conceptual and theoretical papers were included when they provided clinically influential models or clarified mechanisms not adequately captured by outcome studies alone.

The strength of evidence for each proposed failure mode was described qualitatively rather than graded numerically. Mechanisms were characterized as having a strong, moderate, or emerging evidence base depending on the consistency of findings, methodological quality of available studies, proximity to clinical psychotherapy outcomes, and degree of support from experimental or translational literature. Where evidence was indirect, heterogeneous, or primarily theoretical, this was stated explicitly.

### 2.7. Narrative Synthesis

The synthesis proceeded in three stages. First, therapeutic mechanisms relevant to anxiety psychotherapy were identified, including inhibitory learning, cognitive reappraisal, attentional modulation, emotion regulation, avoidance reversal, and interpersonal learning. Second, pathogenic processes that may prevent these therapeutic mechanisms from operating were organized across neurobiological, cognitive-behavioral, and interpersonal-developmental levels. Third, recurrent clinical patterns of non-response were grouped into a maintenance-mechanism-based taxonomy of mechanistic failure modes.

The synthesis deliberately avoided treating psychotherapy schools as fixed explanatory units. Instead, the analysis asked which therapeutic mechanism was expected to operate, whether the patient’s dominant maintaining process was likely to be engaged by that mechanism, and what clinical pattern might be expected when engagement failed. This approach allowed evidence from CBT, exposure therapy, acceptance-based approaches, psychodynamic formulations, fear-learning research, stress physiology, and attachment theory to be integrated without implying that all forms of psychotherapy share identical mechanisms.

### 2.8. Development of the Clinical Taxonomy

The proposed taxonomy was developed through iterative comparison of the extracted evidence with common patterns of psychotherapy non-response described in the clinical and empirical literature. Each failure mode was defined by four linked elements: dominant pathogenic process, disrupted therapeutic mechanism, clinical signature of non-response, and corrective treatment operation. The taxonomy was then examined for disorder-specific expression across panic disorder, agoraphobia, social anxiety disorder, generalized anxiety disorder, specific phobia, separation anxiety disorder presentations, and mixed anxiety states complicated by trauma or chronic stress.

The taxonomy is intended as a formulation aid rather than a diagnostic classification or validated treatment-selection algorithm. It is designed to help clinicians interpret non-response after an apparently adequate course of evidence-based psychotherapy and to generate hypotheses about treatment redesign, sequencing, augmentation, or integration with pharmacotherapy and relational work.

### 2.9. Methodological Boundaries

This review was not designed as a systematic review, scoping review, or meta-analysis. It does not claim exhaustive capture of all available studies, and no pooled estimates of effect size were calculated. The purpose was to provide a structured, clinically grounded synthesis of mechanisms relevant to psychotherapy non-response in anxiety disorders. To reduce the risk of selective interpretation, the search strategy, eligibility criteria, evidence prioritization, and synthesis logic were specified explicitly, and the limitations of the evidence base are addressed separately.

## 3. Mechanisms of Action in Psychotherapy

Psychotherapies are often described by school: CBT, acceptance and commitment therapy, psychodynamic therapy, mindfulness-based therapy, metacognitive therapy, or interpersonal therapy [8,34,42]. This taxonomy is useful for training, research, and reimbursement, but it can obscure mechanisms. Two treatments with different labels may engage overlapping mechanisms, and two patients receiving the same manual may experience different mechanisms depending on formulation, therapist behavior, and context [12,16]. A mechanism-based view is especially important in treatment failure because non-response often occurs when the presumed active process has not been adequately activated [12,14,19].

Exposure therapy is among the most extensively studied psychological interventions for anxiety disorders [8,34]. Early emotional processing theory emphasized activation of the fear structure and incorporation of corrective information incompatible with feared outcomes [12,13,14]. Contemporary models place greater emphasis on inhibitory learning. In this view, exposure does not erase original fear learning. Rather, it creates new safety or non-threat associations that inhibit fear expression under certain conditions [12,13,14,28,43]. This distinction is clinically important. If fear memory is not erased, relapse is not surprising. Return of fear may occur through renewal in a new context, reinstatement after stress or aversive events, spontaneous recovery over time, or reacquisition [12,13,14,28].

Inhibitory learning models emphasize expectancy violation, variability, retrieval cues, removal of safety behaviors, and generalization across contexts [12,13,14,44]. A patient with panic disorder who repeatedly induces dizziness and learns that the sensation can be tolerated without collapse is not simply habituating. The patient is acquiring new predictions about bodily arousal. A patient with social anxiety who asks a question in a meeting and discovers that humiliation does not occur is learning in a social-evaluative context. The clinical target is not fear reduction during the session alone, but durable learning that can be retrieved in the patient’s ordinary environment [12,14,43].

This distinction helps explain some failures of exposure therapy. If exposure is conducted primarily to achieve short-term anxiety reduction, the patient may learn that fear declines only when rituals, reassurance, therapist proximity, or subtle avoidance are present [12,14,37,38]. If exposures are too predictable, narrow, or context-bound, inhibitory learning may remain fragile [12,14,28,43]. If exposure is terminated whenever anxiety peaks, the patient may learn that escape prevents catastrophe [12,14]. Thus, exposure failure is often a failure of learning design rather than a failure of courage [12,14].

Cognitive interventions aim to modify threat appraisals, probability estimates, catastrophic interpretations, and maladaptive beliefs about uncertainty, bodily sensations, social evaluation, or loss of control [8,34,42]. Cognitive restructuring is frequently misunderstood as persuasion or positive thinking. Its more precise mechanism is reappraisal: the generation and testing of alternative interpretations that change the meaning of internal or external stimuli [45]. Neuroimaging meta-analyses of cognitive reappraisal implicate prefrontal and anterior cingulate networks involved in cognitive control and modulation of affective responding [45,46]. In psychotherapy, reappraisal is rarely achieved through verbal disputation alone. It is strengthened by behavioral experiments, corrective experience, and repeated practice under affective activation [8,34,46].

The evidence that threat reappraisal mediates CBT outcomes in anxiety disorders is suggestive but not uniformly definitive. A systematic review concluded that threat reappraisal is a plausible mediator, while noting methodological limitations in mediation research, including temporal ambiguity and inconsistent measurement [45,46]. Clinically, this implies that cognitive change matters, but not all patients can access it in the same way. Some patients can identify catastrophic predictions and test them; others enter a perseverative debate in which each alternative explanation generates new doubt. For these patients, conventional cognitive restructuring may inadvertently reinforce threat monitoring and reassurance seeking [23,24,47].

Anxiety disorders also involve altered allocation of attention. Meta-analytic work supports an association between anxiety and threat-related attentional bias, though effects vary by task, disorder, and experimental conditions [48,49]. Attention may be captured by external threat cues, internal sensations, possible signs of social rejection, or ambiguous future danger. Some patients show vigilance toward threat; others alternate between vigilance and avoidance [48,50]. In social anxiety disorder, self-focused attention and monitoring of perceived visible anxiety are central maintaining processes [51]. In panic disorder, attentional narrowing toward interoceptive sensations can amplify catastrophic misinterpretation. In generalized anxiety disorder, attention may be repeatedly drawn toward uncertain future scenarios [48,50].

Psychotherapy can modify attention in several ways. Exposure may train attention toward feared stimuli without escape [12,14,48,50]. Mindfulness-based approaches may cultivate decentered awareness of internal events [46,51]. Attention training may redirect focus from self-monitoring to task and environment [51]. Cognitive therapy may reduce interpretive bias, thereby changing what is noticed as salient [48,49,50]. When psychotherapy fails, it is often because attentional processes remain unmodified. A patient may complete a social exposure while scanning faces for disapproval, rehearsing sentences internally, and monitoring blush intensity. Behaviorally, the exposure occurred; mechanistically, the patient remained in threat surveillance [48,50].

Emotion regulation is not a single faculty. It includes cognitive reappraisal, attentional deployment, inhibitory control, acceptance, interoceptive tolerance, autonomic regulation, affect labeling, and interpersonal co-regulation [46]. Top-down regulation refers broadly to prefrontal and executive processes that modulate emotional responses through interpretation, attention, and goal-directed control. Bottom-up processes refer to autonomic, interoceptive, limbic, and sensory-affective signals that shape emotion before deliberate reflection is possible [52]. This distinction is clinically useful, although the neurobiology is more integrated than the metaphor implies [52,53].

Many psychotherapies rely substantially on top-down capacities. Cognitive restructuring asks patients to observe thoughts, evaluate evidence, and generate alternatives. Exposure asks patients to inhibit avoidance long enough for new learning to occur. Acceptance and commitment therapy asks patients to notice internal experiences, defuse from thoughts, and act according to values. Psychodynamic therapy asks patients to reflect on affective meanings and relational patterns [8,34,42,53]. When arousal is extreme, sleep is impaired, trauma reminders are active, or stress physiology is dysregulated, these capacities may be compromised [22,25]. Acute stress can impair extinction retrieval and prefrontal-dependent learning [22,25,26]. A patient who cannot access reflective functioning during high arousal may not be unmotivated; the treatment may be demanding a regulatory capacity that is temporarily unavailable [52,53].

Bottom-up interventions do not replace psychotherapy mechanisms, but they may prepare the system to learn. Interoceptive exposure, careful arousal management, grounding, sleep stabilization, exercise, pharmacotherapy, and somatic awareness may reduce the dominance of physiological threat signals [25,52,53]. The risk is that regulation strategies become safety behaviors if used to eliminate anxiety before learning can occur [12,14,37,38]. The therapeutic task is therefore to distinguish regulation that enables learning from regulation that prevents learning [37,38,53].

Finally, psychotherapy is an interpersonal treatment even when the protocol is highly structured. The therapeutic relationship provides safety, expectancy, collaboration, modeling, rupture repair, and sometimes corrective relational experience [32,54,55]. Attachment insecurity, shame, interpersonal trauma, and impaired mentalization can interfere with these processes [32,54,56]. Patients with anxious attachment may experience therapist neutrality as rejection or delayed improvement as abandonment. Patients with avoidant attachment may comply behaviorally while withholding affectively significant material. Patients with disorganized attachment or trauma histories may experience the therapist’s questions, exposure assignments, or interpretations as coercive or dangerous [32,54].

Evidence linking adult attachment to anxiety, especially social anxiety, supports the clinical relevance of relational processes, although causality and treatment-specific mechanisms remain less established than for exposure and CBT [15,32,54]. Psychodynamic and mentalization-informed approaches emphasize how anxiety is embedded in relational expectations, affect avoidance, shame, and self-other representations [34]. CBT also depends on relational mechanisms, particularly alliance, collaboration, and willingness to test predictions [27,32,55]. In treatment failure, the alliance may appear superficially intact while the patient privately experiences fear, compliance, resentment, or withdrawal. Without attention to these interpersonal mechanisms, technical interventions may not reach the pathogenic level at which anxiety is maintained [32,54,56].

## 4. Pathogenesis of Anxiety Disorders: A Multilevel Model

Anxiety disorders cannot be reduced to amygdala hyperactivity, irrational beliefs, avoidance, trauma, attachment insecurity, or stress hormones alone [8,18,29,30]. Each of these levels is relevant, but none is sufficient. A maintenance-mechanism-based framework requires a multilevel model in which neurobiological vulnerability, cognitive-affective processing, learning history, behavior, and interpersonal context interact over time [8,16,18,19]. The clinical expression of anxiety is therefore not a simple output of one defective system. It is a dynamic state in which threat detection, prediction, regulation, avoidance, and relational meaning become mutually reinforcing [8,18,29,30].

At the neurobiological level, functional neuroimaging studies implicate distributed threat and salience networks in anxiety disorders. Meta-analytic work has identified altered activation in the amygdala, insula, anterior cingulate, and prefrontal regions across anxiety-related conditions, although findings differ by disorder, task, medication status, comorbidity, and analytic method [18,29,30]. The amygdala is central to threat detection and fear learning, but it is not a simple fear center. It interacts with sensory, hippocampal, prefrontal, striatal, and autonomic systems [29,30]. The insula is important for interoception and anticipation of aversive bodily states. The hippocampus contributes to contextual learning and memory. The anterior cingulate and prefrontal cortices participate in conflict monitoring, appraisal, extinction learning, and regulation [29,30].

Amygdala hyperreactivity is commonly discussed in anxiety disorders, especially in relation to threat cues and emotional faces. However, the evidence is heterogeneous [18,29,30]. Some patients show exaggerated amygdala responses; others show abnormalities in connectivity, salience processing, or prefrontal regulation rather than simple hyperactivation [29,30]. Social anxiety disorder, for example, has been associated with altered responses to social-evaluative stimuli in amygdala and related networks, but meta-analytic reviews emphasize broader neurofunctional models rather than isolated regional abnormalities. This caution matters clinically. If anxiety is conceptualized too narrowly as amygdala overactivity, psychotherapy failure may be misunderstood as insufficient fear reduction rather than as failure of contextual learning, attention, or interpersonal safety [12,29,30].

Prefrontal regulatory deficits are also best understood as network-level alterations. Ventromedial prefrontal cortex is implicated in extinction recall and safety learning; dorsolateral prefrontal cortex in cognitive control; dorsal anterior cingulate in threat appraisal, conflict, and action selection [29,30]. In anxiety disorders, impaired regulation may manifest as difficulty using contextual safety information, inability to disengage from threat, reduced cognitive flexibility, or failure to inhibit avoidance [30]. These processes are directly relevant to psychotherapy because many treatments require patients to tolerate threat activation while acquiring new predictions [12]. A treatment that assumes intact cognitive flexibility may fail when prefrontal control is compromised by stress, insomnia, depression, or developmental adversity [22,25,52].

The hypothalamic–pituitary–adrenal (HPA) axis is another relevant but complex system. Cortisol and stress reactivity have been studied extensively across psychiatric disorders. Meta-analytic evidence suggests altered cortisol stress responses in psychiatric populations, but findings in anxiety disorders are not uniform. Basal cortisol, awakening response, diurnal slope, and stress reactivity may differ by diagnosis, chronicity, trauma exposure, medication, sleep, sex, and developmental history. It would be inaccurate to treat HPA dysregulation as a diagnostic biomarker for anxiety disorders. Clinically, however, stress-system activation may influence treatment by affecting sleep, attention, arousal, extinction learning, and memory consolidation [22,25,26].

At the cognitive-behavioral level, anxiety disorders are maintained by threat bias, catastrophic interpretation, intolerance of uncertainty, anxiety sensitivity, worry, safety behaviors, and avoidance [8,34,42]. Threat-related attentional bias is associated with anxiety, although its magnitude and clinical utility vary [57]. Catastrophic misinterpretation is especially prominent in panic disorder, where benign bodily sensations are interpreted as signs of imminent catastrophe [8,34]. In social anxiety disorder, ambiguous social cues are interpreted as evidence of negative evaluation. In generalized anxiety disorder, uncertain future events are experienced as intolerable and requiring mental preparation or worry [8,23,24,34,47]. These processes are often accessible to clinical observation, which makes them attractive targets for formulation and treatment adaptation [16,19].

Intolerance of uncertainty is one of the most important transdiagnostic constructs in anxiety. Meta-analytic evidence supports its association with worry and anxiety symptoms [23,24,47]. It is not simply a preference for predictability; it involves a dispositional and learned difficulty tolerating the possibility of negative events, even when probability is low [23,47]. Patients high in intolerance of uncertainty often seek reassurance, over-prepare, check, avoid decisions, or engage in worry as a form of attempted control [23,24,47]. Cognitive interventions that focus only on probability estimation may be insufficient if the patient’s core problem is not exaggerated probability but inability to tolerate unresolved possibility [24,47].

Avoidance is the behavioral engine of anxiety chronicity. It includes overt avoidance, escape, reassurance seeking, checking, safety behaviors, emotional suppression, rumination, worry, and interpersonal withdrawal [37,38]. Avoidance reduces distress in the short term and is therefore negatively reinforced. Over time, it prevents corrective learning, narrows functioning, and confirms perceived vulnerability [37]. Contemporary accounts increasingly view avoidance not merely as a symptom but as a central process linking threat appraisal, reinforcement, habit formation, and functional impairment [36,37,38]. Psychotherapy succeeds when it modifies avoidance sufficiently for new learning and valued action to occur [12,37,38].

The interpersonal and developmental level gives these processes personal meaning. Developmental adversity, trauma, attachment insecurity, and relational learning can shape anxiety pathogenesis [32,54,58]. Early adversity is associated with increased risk for later mental disorders and with alterations in stress responsivity, emotional learning, and threat processing. These associations are not deterministic. Many individuals exposed to adversity do not develop anxiety disorders, and many patients with anxiety disorders do not report major trauma. Nonetheless, developmental context matters because it shapes expectations about safety, control, attachment, shame, and the reliability of others [54,58].

Attachment processes are particularly relevant in social anxiety, separation anxiety, panic with dependency fears, and generalized anxiety embedded in interpersonal responsibility [32,54,58]. Insecure attachment may increase vigilance to rejection, difficulty seeking support, fear of abandonment, or compulsive self-reliance [54,58]. Mentalization, the capacity to understand oneself and others in terms of mental states, may be compromised under stress or in the context of trauma. When mentalization collapses, ambiguous interpersonal cues can be experienced as immediate evidence of threat. In therapy, this may appear as sudden withdrawal, appeasement, mistrust, or intense fear of therapist judgment [32,54,58].

These developmental and interpersonal mechanisms do not replace neurobiological and cognitive-behavioral models. They provide the learning history through which threat systems, beliefs, and avoidance strategies acquire personal meaning [54,59]. A patient may avoid meetings not only because of exaggerated probability of embarrassment, but because early experiences taught that visible vulnerability leads to humiliation. Another may fear panic sensations not only because of catastrophic bodily misinterpretation, but because loss of control in attachment relationships has historically been dangerous. Mechanistic formulation must therefore ask not only what the patient fears, but what level of the system is maintaining the fear [8,16,18,19].

## 5. Why Psychotherapy Fails: Mechanistic Failure Modes

Psychotherapy failure in anxiety disorders is often multifactorial. Poor access, inadequate dose, therapist inexperience, comorbid depression or substance use, ongoing stressors, medication effects, and socioeconomic constraints can all contribute [8,10,34]. The framework proposed here focuses on a specific subset of clinically important failures: those arising when the therapy’s active mechanism does not match the patient’s dominant pathogenic mechanism. Six failure modes are described below. They are not mutually exclusive. In practice, patients often present with combinations, and the task is to identify which mechanism currently prevents therapeutic learning [12,13,19]. The six proposed failure modes are summarized in Table 2. The table links each mode to its dominant maintaining process, strength of evidence, typical clinical signature, and mechanism-based treatment adaptation.

The first failure mode is impaired fear extinction or inhibitory learning. The underlying mechanism is insufficient acquisition, consolidation, retrieval, or generalization of inhibitory safety learning [12,13,28,43,60]. The patient may complete exposures but fail to develop durable non-threat associations. This can occur when exposures are too brief, too predictable, too reliant on within-session habituation, too dependent on therapist presence, or contaminated by safety behaviors [12,36,37,43]. It can also occur when exposures do not target the patient’s actual expectancy [12,43]. A patient afraid of fainting during panic may repeatedly enter crowded places but avoid inducing the feared sensations. A patient with social anxiety may attend gatherings but speak only when rehearsed, avoid eye contact, and leave early. The feared prediction remains untested [12,43]. The evidence base for this failure mode is strong at the level of fear learning and exposure principles. Experimental and clinical literature shows that extinction is context-dependent and vulnerable to return of fear [12,13,28,43,60,61]. Translational work supports the importance of expectancy violation, variability, retrieval cues, and reduced safety behaviors [12,28,43,44]. However, the exact clinical prediction of individual exposure failure remains less precise. Not every patient who relapses has impaired extinction; relapse may reflect renewed stress, new learning, medication changes, interpersonal conflict, or life context [40,41]. The framework therefore treats impaired inhibitory learning as a testable clinical hypothesis rather than a universal explanation [12,43]. Clinically, impaired inhibitory learning is suggested when the patient reports that exposure was completed but did not change belief, when gains occur only in the therapist’s office, when fear returns immediately in new contexts, or when the patient relies on subtle safety behaviors [12,36,43]. The therapeutic implication is not simply more exposure, but better-designed exposure. The clinician should identify the feared expectancy, remove safety behaviors gradually but deliberately, vary contexts, introduce occasional high-intensity or unpredictable exposures, use retrieval cues, and emphasize learning rather than anxiety reduction [12,43,44]. For some patients, exposure should be framed as a laboratory for testing predictions rather than an endurance exercise [12,43].

The second failure mode is cognitive rigidity and reappraisal failure. Cognitive rigidity refers to difficulty revising threat beliefs despite evidence, difficulty tolerating ambiguity, perseverative worry, and overvaluation of certainty [47,62,63]. In this failure mode, psychotherapy fails because the patient can understand alternative interpretations intellectually but cannot use them flexibly under uncertainty [23,24,47,63]. The patient may respond to every cognitive intervention with a new objection or demand for further certainty. The problem is not lack of intelligence; indeed, many such patients are highly verbally skilled. The problem is that verbal reasoning is recruited into threat management, producing more analysis, more reassurance seeking, and more uncertainty monitoring [23,24,47]. The evidence supporting this failure mode comes from research on intolerance of uncertainty, worry, cognitive reappraisal, and metacognitive processes [23,24,63]. Generalized anxiety disorder is the paradigmatic example, but cognitive rigidity also appears in panic disorder, social anxiety disorder, health anxiety, and obsessive-compulsive phenomena [23,24,47]. Within OCD itself, dysfunctional metacognitive belief profiles are not homogeneous across symptom dimensions; recent systematic evidence suggests that different metacognitive domains map differentially onto checking, unacceptable thoughts, symmetry/ordering, contamination/washing, and hoarding presentations, supporting dimension-informed formulation and converging with the mechanism-matched logic proposed here. Conventional cognitive restructuring can be helpful when distorted probability estimates are central. It may be less effective when the pathogenic process is the demand for certainty itself [23,24,47]. In that case, repeated attempts to prove safety may reinforce the premise that safety must be proven before action [24,47]. Clinically, cognitive rigidity is suggested when therapy sessions become repetitive debates, when reassurance produces transient relief but no durable change, when behavioral experiments are interpreted narrowly, or when the patient insists on eliminating doubt before acting [23,24,47,63]. The therapeutic implication is to shift from content correction to process change. Interventions may include uncertainty exposure, behavioral experiments that test tolerance rather than probability, metacognitive work on worry as a strategy, acceptance-based diffusion, and deliberate reduction in reassurance [23,24,47]. The aim is not to convince the patient that feared outcomes are impossible, but to help the patient act without impossible certainty [23,24,47].

The third failure mode is stress-induced learning impairment. Some patients enter psychotherapy in a state of chronic hyperarousal, sleep disruption, ongoing threat, or high physiological stress [21,25,64,65]. In such cases, therapy may fail because stress impairs the learning and memory processes on which psychotherapy depends. Acute stress has been shown experimentally to affect extinction learning and retrieval, and animal and human studies indicate that stress can alter prefrontal-amygdala dynamics relevant to fear regulation [21,25,64,65]. HPA-axis findings in anxiety disorders are heterogeneous, but clinically, severe stress load can impair attention, working memory, emotion regulation, and consolidation [25,65,66,67]. This failure mode is particularly relevant when anxiety is complicated by trauma exposure, unstable living conditions, caregiving burden, occupational threat, medical illness, insomnia, or recurrent panic [21,25,66,67]. The patient may attend sessions but be unable to encode new learning. Exposure may be experienced as flooding rather than corrective learning. Cognitive restructuring may be inaccessible because the patient is exhausted or physiologically overwhelmed [21,25,64,66,67]. The therapist may misinterpret this as avoidance or poor motivation, when the immediate problem is that the patient’s neurophysiological state is not conducive to learning [21,25,67]. Clinical signs include poor recall of session content, marked dissociation or shutdown during exposure, worsening sleep after sessions, inability to complete homework despite agreement, and rapid return of fear after acute stressors [21,25,67]. The implication is not to avoid therapeutic challenge indefinitely. Rather, treatment may require sequencing. Sleep stabilization, reduction in acute stressors where possible, pharmacotherapy for severe anxiety or comorbid depression, skills for tolerating arousal, and careful titration of exposure may be necessary before intensive expectancy violation can consolidate [21,25,66]. The clinician should avoid both extremes: premature high-intensity exposure that overwhelms learning, and endless stabilization that becomes avoidance [12,21,25,26].

The fourth failure mode is attentional dysregulation. In attentional dysregulation, therapy fails because the patient’s attention remains locked onto threat signals even while outwardly engaging in treatment tasks [48,50,51,68,69,70]. A patient with social anxiety may complete a conversation exposure while monitoring facial expressions for disapproval and tracking internal signs of blushing [68,69,70]. A patient with panic disorder may drive on the highway while continuously scanning heart rate and breathing. A patient with generalized anxiety disorder may practice relaxation while monitoring whether relaxation is working and whether future danger has been adequately anticipated [48,50]. The evidence base includes studies of threat-related attentional bias, self-focused attention, interoceptive monitoring, and attentional control [49,68,69,70]. The findings are not sufficiently consistent to support simple attentional-bias tests as routine treatment-selection tools, but they are clinically informative [48,49,50]. Attention determines what information is available for learning. If the patient’s attentional field is restricted to threat-confirming cues, corrective information may not be encoded [48,68,69]. A social exposure that is remembered only as a sequence of internal anxiety sensations is not equivalent to an exposure in which the patient processes the actual responses of others [51,68,69,70]. Clinically, attentional dysregulation is suggested when patients report persistent fear despite repeated behavioral engagement, when they cannot recall neutral or positive information from exposures, or when they describe sessions in terms of internal monitoring rather than external learning [68,69,70]. Treatment adaptations include attention training, external focus exercises, mindfulness of bodily sensations without threat interpretation, interoceptive exposure, video feedback for social anxiety, and explicit practice broadening attention during exposure [48,50,51]. The goal is not distraction from anxiety, which can become avoidance, but flexible attention that allows feared cues, safety cues, and task-relevant information to be processed [48,50,51].

The fifth failure mode is attachment-related barrier formation. Attachment-related failure occurs when the interpersonal conditions required for psychotherapy are disrupted by mistrust, shame, fear of dependency, fear of criticism, or relational expectations derived from earlier learning [15,71,72,73]. The patient may appear non-adherent, guarded, excessively compliant, provocative, or disengaged. The mechanism is not simply poor alliance. It is the therapeutic relationship itself that activates threat [15,32,54,59,71,72,73,74]. The therapist’s curiosity may feel intrusive; exposure assignments may feel coercive; cognitive questioning may feel invalidating; neutrality may feel abandonment; praise may feel manipulative [32,72,73,74]. Evidence for attachment-related barriers is less experimentally mature than the evidence for exposure mechanisms, but associations between attachment insecurity, social anxiety, interpersonal impairment, and mental health are clinically meaningful [72,73,74]. Psychotherapy process research across disorders also supports the importance of alliance and rupture repair, although alliance is both a common factor and a potential marker of early improvement [32,56,71,74]. The mechanistic claim is therefore made cautiously: attachment processes may interfere with psychotherapy when they prevent collaboration, emotional disclosure, corrective relational learning, or the patient’s ability to use the therapist as a secure base [71,72,74]. Clinically, attachment-related barriers are suggested by repeated missed sessions after perceived criticism, excessive agreement without affective engagement, sudden withdrawal after closeness, intense shame about symptoms, or recurrent enactments of rejection and control [15,71,72,74]. Treatment implications include explicit attention to the therapeutic frame, careful rupture repair, collaborative formulation, validation before challenge, and attention to shame and interpersonal meaning [27,56,75]. For some patients, psychodynamic, interpersonal, schema-focused, or mentalization-informed work may be necessary before or alongside exposure and cognitive interventions [34,71,73]. This does not mean abandoning evidence-based methods; it means creating the relational conditions under which those methods can operate [32,71,72,73].

The sixth failure mode is chronic avoidance dominance. Avoidance dominance occurs when avoidance has become the organizing principle of the patient’s life [36,37,38,39]. The patient may avoid places, sensations, uncertainty, conflict, intimacy, decisions, responsibility, or emotional states. Avoidance may be overt, but it is often covert: reassurance seeking, over-preparation, mental rehearsal, checking, emotional numbing, substance use, excessive sleep, perfectionism, or dependence on companions [36,37,38,39]. In this failure mode, psychotherapy fails because avoidance is more immediately reinforcing than therapeutic learning [36,37,39]. The evidence that avoidance maintains anxiety is foundational across behavioral and cognitive-behavioral models [13,36,37,38,39,53]. Yet chronic avoidance dominance is clinically distinct from ordinary avoidance. It often reflects years of reinforcement, family accommodation, occupational adaptation, and identity-level narrowing [76]. A patient may agree that avoidance maintains anxiety while remaining unable to relinquish it because avoidance protects employment, relationships, self-esteem, or a fragile sense of control [37,39,76]. In such cases, exposure homework may fail not because the patient does not understand it, but because the contingencies outside therapy overwhelmingly reward avoidance [37,38,39,76]. Clinical signs include repeated failure to complete between-session tasks, extensive safety behaviors, family members facilitating avoidance, narrowing of life activities, and statements that indicate knowing what needs to be done without being able to initiate it [36,39,76]. Treatment implications include detailed functional analysis, motivational interviewing, contingency planning, reduction in family accommodation, values-based activation, and graded exposure embedded in meaningful goals [37,38,39,76]. The clinician must identify what avoidance accomplishes for the patient. Only then can treatment build alternative sources of reinforcement strong enough to compete with avoidance [37,38,76].

The evidentiary support for these six failure modes is uneven. Some categories, particularly impaired inhibitory learning and chronic avoidance dominance, are supported by stronger behavioral and translational studies, whereas attachment-related barriers and stress-related learning impairment remain more dependent on convergent clinical and translational evidence. The qualitative strength of evidence for each proposed failure mode is summarized in Table 3. To avoid overstating the taxonomy as a validated precision-treatment algorithm, Table 3 summarizes the qualitative strength of evidence supporting each failure mode and the main limitations of the current evidence base.

The weaker evidence categories should therefore be interpreted with particular caution. Attachment-related barriers and stress-related learning impairment are clinically plausible and supported by convergent evidence from psychotherapy process research, attachment theory, stress physiology, sleep research, and fear-learning studies, but they are not yet validated treatment-matching categories. Their inclusion in the taxonomy is intended to guide careful formulation and hypothesis testing rather than to justify automatic treatment allocation. Relational-first adaptations, stress-sequenced exposure, and other mechanism-specific modifications should be evaluated in pilot studies and controlled trials using mechanism-level monitoring before they are recommended for broad routine implementation.

**Figure 1:**
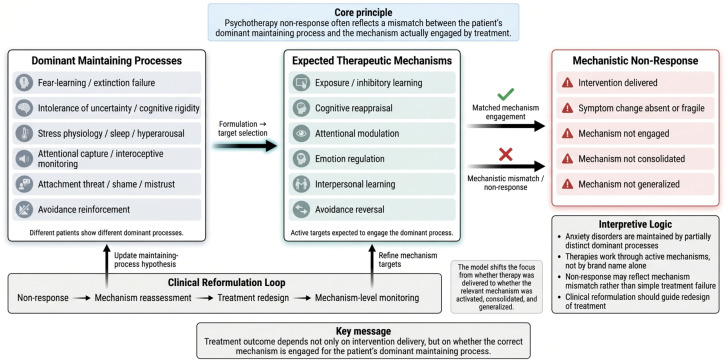
Integrative model linking pathogenesis, therapeutic mechanisms, and psychotherapy outcome. Multilevel pathogenesis, encompassing neurobiological vulnerability, cognitive-behavioral maintenance, and interpersonal-developmental learning, converges on a dominant maintaining mechanism in the individual patient. Psychotherapy is most effective when its active mechanism engages this dominant process. Adequate mechanism engagement promotes new learning, generalization, symptom reduction, functional recovery, and relapse resilience. By contrast, mechanism mismatch may result in pseudo-engagement, incomplete learning, dropout, relapse, or persistent symptoms. Failed outcomes may further increase threat salience and avoidance, thereby reinforcing the maintenance mechanism.

The integrative logic linking multilevel pathogenesis to psychotherapy outcome is summarized in Figure 1. In this model, neurobiological vulnerability, cognitive-behavioral maintenance, and interpersonal-developmental learning converge on a dominant maintaining mechanism. Psychotherapy is expected to produce durable improvement when its active mechanisms engage this dominant process; when they do not, apparent participation in treatment may coexist with incomplete learning, relapse, dropout, or persistent symptoms.

## 6. A Maintenance-Mechanism-Based Framework for Treatment Matching

The proposed taxonomy rests on a clinical premise: after non response, treatment selection should be reconsidered not only in relation to diagnosis, but also in relation to the mechanism most likely to be maintaining the patient’s anxiety at that time [10,19,34]. Diagnosis remains clinically necessary. It guides differential diagnosis, risk assessment, psychoeducation, and initial treatment selection [8,34]. However, DSM or ICD categories do not specify which mechanism is dominant in a given patient [16,19]. Two patients with panic disorder may require different interventions: one may need interoceptive exposure for catastrophic misinterpretation of bodily sensations; another may need attachment-focused work because panic occurs primarily in contexts of separation and dependency threat [12,32,54,73]. Two patients with generalized anxiety disorder may both worry constantly, but one may be driven by intolerance of uncertainty, another by trauma-related hypervigilance, and another by family accommodation and avoidance of conflict [23,24,31].

The maintenance-mechanism-based model links four elements: pathogenic process, disrupted therapeutic mechanism, therapy mismatch, and alternative strategy [10,19]. Psychotherapy may fail when treatment emphasizes a mechanism that is not currently accessible, not central to the patient’s presentation, or insufficiently activated during treatment [10,12,19]. For example, a cognitive therapy emphasizing verbal reappraisal may mismatch a patient whose primary impairment is fear extinction and interoceptive avoidance [12]. Conversely, intensive exposure may mismatch a patient whose dominant barrier is attachment-related mistrust and shame, because the patient may experience exposure assignments as relational threat and disengage [58,73]. Relaxation-based intervention may reduce arousal temporarily but mismatch a patient whose anxiety is maintained by avoidance and intolerance of uncertainty [23,47]. Psychodynamic exploration may increase insight but mismatch a patient whose phobic avoidance requires direct behavioral learning [12]. Table 4 presents a maintenance-mechanism-based treatment matching model, linking dominant maintaining processes to the therapeutic mechanisms that failed to engage, common clinical mismatches, and mechanism-matched adaptations.

This framework is not an argument for eclecticism without discipline. Treatment matching must remain anchored in evidence-based methods [8,10,34]. The question is not which therapy the clinician prefers, but which mechanism must change. If the dominant process is avoidance and catastrophic expectancy, exposure-based CBT remains central [12,68]. If the dominant process is intolerance of uncertainty, CBT should explicitly target uncertainty through behavioral experiments and reduction in reassurance, rather than merely challenge worry content [47,63]. If the dominant process is attentional self-monitoring, therapy should include external focus and attention flexibility [48,50,68,69]. If the dominant process is stress-induced learning impairment, treatment may need sequencing and biological support before intensive learning tasks [25,77]. If the dominant process is attachment-related threat, relational safety and rupture repair may be prerequisites for effective cognitive or behavioral work [32,54,73,78].

Because failure modes often co-occur, the treatment-matching model should be applied sequentially rather than as a static classification. A pragmatic rule is to prioritize the process that most immediately prevents therapeutic learning. When severe insomnia, marked hyperarousal, dissociation, acute environmental threat, uncontrolled comorbidity, substance use, or sedative medication effects are prominent, stress-related learning impairment should usually be addressed before redesigning exposure, because corrective learning may not be reliably encoded, consolidated, or retrieved under unstable learning conditions. Once basic learning conditions are adequate, the next target should be the residual failure mode with the clearest clinical signature: untested feared expectancy or persistent safety behaviors suggest impaired inhibitory learning; reassurance-driven inability to act under doubt suggests cognitive rigidity or intolerance of uncertainty; persistent self-monitoring or threat scanning suggests attentional dysregulation; shame, mistrust, or rupture sensitivity suggests attachment-related barriers; and repeated failure of between-session implementation despite agreement suggests chronic avoidance dominance.

The translational relevance of the framework lies in its bridge between neuroscience and clinical formulation. Fear-learning research suggests that exposure should be designed to maximize inhibitory learning [13,60,79,80,81]. Stress neuroscience suggests that high arousal and sleep disruption can impair learning and retrieval [25,64,82]. Cognitive science suggests that intolerance of uncertainty and attentional bias may maintain symptoms even when feared outcomes do not occur [48,50]. Attachment and developmental research suggests that interpersonal threat can determine whether the patient can use the therapist and the treatment frame [32,54,83]. These domains do not yield a simple biomarker algorithm, but they do support a more precise clinical logic [8,10].

Although the proposed taxonomy is transdiagnostic, its clinical expression differs across anxiety disorders. The same failure mode may appear in different forms depending on the feared stimulus, the dominant safety behavior, and the type of learning expected from treatment [12,37]. Panic disorder, generalized anxiety disorder, social anxiety disorder, agoraphobia, and specific phobia may therefore share broad mechanisms such as avoidance and threat learning, but they often differ in the mechanism most likely to block therapeutic change [8,16,19,34]. Table 5 summarizes disorder-specific expressions of mechanistic non-response and links each clinical presentation to likely failure modes, typical signatures of non-response, and corrective treatment implications.

Consider a patient with panic disorder who has attended ten sessions of structured CBT, including psychoeducation and cognitive restructuring, but without systematic interoceptive exposure or explicit testing of feared bodily predictions [12,79,84]. The patient understands that panic attacks are not dangerous but continues to avoid exercise, crowded trains, and driving. The mismatch is between a verbally mediated intervention and an interoceptive fear-learning problem. Treatment should shift toward interoceptive exposure, behavioral experiments targeting feared bodily sensations, and reduction in safety behaviors such as carrying unnecessary medical devices or repeatedly checking pulse [37,81,84]. The pathogenic process is catastrophic interoceptive prediction; the disrupted therapeutic mechanism is inhibitory learning; the corrective strategy is exposure designed around bodily expectancy violation [12,80,81].

Consider a patient with generalized anxiety disorder who has completed standard CBT worksheets but remains consumed by future uncertainty [47,63]. Each cognitive alternative becomes a new object of doubt. The mismatch is between content-level cognitive restructuring and a process-level intolerance of uncertainty [24,47,63]. Treatment should shift toward uncertainty exposure, scheduled reduction in reassurance, behavioral experiments in making decisions without complete information, and metacognitive work on worry as an attempted control strategy [24,63,85]. The therapeutic aim is not to prove that feared events will not happen; it is to restore functioning in the presence of unresolved possibility [23,24,31].

Consider a patient with social anxiety disorder and a developmental history of humiliation and emotional neglect [83,86]. The patient completes exposures but experiences the therapist’s feedback as criticism and misses sessions after perceived disappointment. The mismatch is between technically correct exposure assignments and unaddressed relational threat [32,54,73]. Treatment should explicitly formulate shame, attachment expectations, and rupture sensitivity [32,54]. Exposure may continue, but it must be embedded in a collaborative frame in which the patient can mentalize the therapist’s intentions and tolerate interpersonal learning [78,83,86]. Here, the mechanism is not only fear extinction but corrective relational learning [32,54,73,83].

Consider a patient with agoraphobic avoidance, severe insomnia, chronic occupational stress, and frequent benzodiazepine use [22,25,77]. Intensive exposure may be necessary eventually, but early attempts may fail because the patient cannot consolidate learning and uses medication as a safety signal [22,37,38]. Treatment may require coordination with pharmacotherapy, sleep intervention, careful benzodiazepine review, and graded exposure designed to prevent overwhelming arousal [22,25,66]. The mismatch is between a high-demand learning intervention and a dysregulated stress-learning system. The corrective strategy is sequencing, not avoidance of exposure [22,77].

The framework also has implications for how clinical progress is evaluated. Symptom reduction is important, but it is not synonymous with mechanism change [87,88]. A patient may report lower anxiety because situations are being avoided more efficiently. A patient may score better on a questionnaire while continuing to rely on reassurance, companions, medication timing, or digital checking as safety behaviors [36,39]. Conversely, symptoms may transiently increase during effective exposure or uncertainty work [24,31,47]. Mechanism-based outcome monitoring therefore requires tracking avoidance, safety behaviors, expectancy change, attentional flexibility, uncertainty tolerance, sleep, and relational engagement alongside symptom severity [88,89].

The model is deliberately iterative. Initial formulation should generate a treatment hypothesis; early treatment data should test it [16,87,89]. If the presumed mechanism changes and symptoms improve, the formulation is supported. If the patient does not improve, the clinician should not simply repeat the same intervention with greater insistence [10,16,19]. The question becomes whether the mechanism was correctly identified, whether the intervention engaged it, whether another mechanism is blocking learning, and whether contextual contingencies outside therapy are maintaining the disorder [10,16,19]. Non-response becomes clinically informative rather than demoralizing [10].

When several failure modes co-occur, the dominant mode should be identified pragmatically as the process that most immediately prevents therapeutic learning from occurring. Marked insomnia, hyperarousal, dissociation, or unstable stress load should usually be prioritized first, because these states can compromise encoding, consolidation, and retrieval of corrective learning before exposure or cognitive work has a fair opportunity to operate. Once basic learning conditions are adequate, the next target should be the residual mechanism with the clearest clinical signature, such as untested expectancy and safety behaviors, reassurance-driven intolerance of uncertainty, persistent threat monitoring, relational threat, or external avoidance reinforcement. Thus, poor exposure generalization in the presence of severe sleep-related hyperarousal would first suggest sequencing to restore tolerable learning conditions, followed by exposure redesign to strengthen inhibitory learning.

### Testable Propositions Generated by the Framework

This framework yields several empirically testable propositions. First, patients whose exposure failure is characterized by persistent safety behaviors and poor expectancy violation should show greater improvement after exposure redesign than after simple exposure dose intensification [36,37,80,84]. Second, patients whose cognitive therapy failure is characterized by reassurance seeking and intolerance of uncertainty should benefit more from uncertainty exposure and metacognitive interventions than from further probability-focused cognitive restructuring [63,85]. Third, patients with high stress load, insomnia, or dissociative shutdown during exposure should show better learning after sequencing interventions that improve arousal regulation and sleep before intensive exposure [25,77,90]. Fourth, patients with social anxiety who complete exposures while maintaining self-focused attention should show greater improvement when attentional flexibility and external-focus procedures are added [48,50,91]. Fifth, patients whose non-response is associated with rupture sensitivity, shame, or mistrust should show improved retention and treatment engagement when relational formulation and rupture repair are explicitly integrated before or alongside exposure-based work [32,83,92].

## 7. Biomarkers and Predictors of Psychotherapy Response

The aspiration of precision psychiatry is to predict which patient will respond to which treatment [93,94]. In anxiety disorders, this aspiration is scientifically legitimate but clinically incomplete. Biomarker studies have examined neuroimaging, psychophysiology, cortisol, genetics, cognitive markers, and symptom profiles [94,95]. The evidence is promising but not yet sufficient for routine individual-level treatment assignment [93,94,95]. A balanced interpretation avoids both nihilism and premature clinical translation. Biomarkers may illuminate mechanisms before they become reliable treatment-selection tools [95,96].

Neuroimaging studies suggest that pretreatment activation in salience, interoceptive, fronto-limbic, and prefrontal networks may predict CBT response in anxiety-related disorders. A systematic review of neurobiological markers concluded that such markers have potential for stratified treatment but require stronger validation before clinical application [95]. A later meta-analysis of task-based fMRI studies found that activation in fronto-insular and dorsomedial prefrontal/dorsal anterior cingulate regions was associated with CBT outcome across anxiety-related disorders [97]. These findings are conceptually important because they converge with the mechanisms discussed above: interoception, salience detection, threat appraisal, and cognitive control [97,98,99].

However, these findings do not yet justify scanning patients to determine who is most likely to benefit from CBT [94,95]. Samples remain modest, tasks vary, analytic pipelines differ, and external validation is limited [95,97]. Many studies use group-level prediction, whereas clinical decisions require individual-level calibration [93,94]. Neuroimaging may eventually help distinguish patients whose anxiety is dominated by interoceptive salience, contextual safety-learning deficits, or prefrontal regulatory constraints [97,99]. At present, it is more defensible to treat imaging findings as mechanistic evidence than as a clinical allocation instrument [94,95].

Cortisol and HPA-axis measures are also plausible predictors, particularly because stress can affect learning and extinction [100,101]. Yet the evidence remains mixed. A systematic review and meta-analysis of cortisol as a predictor of psychological therapy response in anxiety disorders found limited and inconsistent evidence [100]. Cortisol may be more clinically meaningful when considered dynamically in relation to sleep, chronic stress, trauma exposure, medication, and session timing than as a single baseline biomarker [100,101,102]. The absence of a simple cortisol predictor should not obscure the clinical reality that severe stress load can impair therapeutic learning [100,103].

Cognitive markers may be closer to clinical utility. Intolerance of uncertainty, anxiety sensitivity, threat interpretation, attentional bias, avoidance, and safety behaviors are measurable and directly linked to treatment targets [23,47,62]. Their advantage is that they can be assessed repeatedly and translated into formulation [23,47]. Their limitation is that many are not yet validated as formal treatment-selection predictors [47,62]. A patient high in intolerance of uncertainty may benefit from uncertainty-focused CBT, but the field lacks definitive algorithms specifying thresholds, combinations, and sequencing [23,47]. Similarly, attentional bias measures have variable reliability, and their clinical use remains uncertain [47,104].

Clinical predictors such as severity, comorbidity, baseline depression, functional impairment, personality pathology, and early treatment response are relevant but nonspecific [94,105]. Early improvement is one of the most pragmatic indicators in psychotherapy. If a patient shows no meaningful change after an adequate early phase, the clinician should reassess the formulation rather than continue the same procedures unchanged [105,106]. Personalized treatment research has emphasized moderators, but systematic reviews indicate that the field is still far from a mature evidence base for individualized psychotherapy selection [93,94].

The most defensible current position is that biomarkers and predictors should inform hypotheses, not dictate treatment [93,94,95]. Neuroimaging may identify circuits relevant to response; cortisol may index stress-learning conditions; cognitive markers may guide case formulation; digital phenotyping may reveal avoidance patterns [107,108]. None currently replaces clinical assessment [93,94,95]. The immediate clinical task is mechanism-informed measurement-based care: assess the processes presumed to maintain anxiety, monitor whether they are changing, and adapt treatment when they are not [109].

## 8. Clinical Implications

The practical value of the taxonomy lies in distinguishing non-response from nonspecific treatment failure [10,110]. A patient who does not improve after psychotherapy may have received too little treatment, may have had inadequate access, or may have discontinued prematurely [10,34]. These are important but different problems. The present framework applies most directly when an apparently adequate intervention has been delivered, yet the expected therapeutic learning has not occurred [10,110]. In such cases, the clinical task is to identify whether the failure reflects an untested expectancy, persistent safety behavior, intolerance of uncertainty, attentional capture, impaired learning under stress, attachment-related threat, or avoidance contingencies outside the treatment room [10,110]. This is the point at which the taxonomy adds information beyond diagnosis, treatment brand, or general calls for personalized care.

The maintenance-mechanism-based taxonomy may help clinicians interpret non-response with greater specificity [110]. To support clinical translation, Figure 1 and Table 2, Table 3, Table 4 and Table 5 are intended to function as standalone formulation aids, and a one-page clinician rapid guide summarizing failure signatures and first-line adaptations is provided as Supplementary Table S3. In addition to asking about adherence, dose, comorbidity, and treatment fidelity, the clinician can ask whether the treatment engaged the mechanism most likely to be maintaining the disorder [10,110]. This shift has practical implications for assessment, treatment planning, sequencing, combined treatment, and supervision [87]. It also has ethical significance. The language of resistance can imply blame, even when clinicians do not intend it. A mismatch formulation preserves responsibility for therapeutic work while reducing moral judgment toward the patient.

Initial assessment should include diagnosis, risk, comorbidity, and impairment, but also a mechanism map [8,34]. The clinician should assess fear predictions, avoidance patterns, safety behaviors, intolerance of uncertainty, attentional focus, interoceptive fear, stress load, sleep, trauma history, attachment patterns, family accommodation, and previous treatment mechanisms [8]. It is not enough to know that the patient had CBT. The clinician must know what CBT consisted of [8,111]. Did it include exposure? Were safety behaviors addressed? Were feared predictions identified? Was uncertainty targeted? Were relational ruptures repaired? Was the patient physiologically able to learn? [32].

Personalized psychotherapy does not mean inventing a new therapy for each patient [34]. It means selecting and sequencing evidence-based components according to mechanism [10,110]. For many patients, standard disorder-specific CBT will be appropriate [34]. For others, the protocol must be adapted. A patient with panic disorder may need more interoceptive exposure and less verbal reassurance [34]. A patient with generalized anxiety disorder may need direct uncertainty work rather than repeated probability analysis [10,110]. A patient with social anxiety disorder may need attention training and video feedback before or during exposure [32]. A patient with attachment-related shame may need relational stabilization and rupture repair before intensive behavioral challenge [32,112].

Sequencing is central. Some mechanisms must be addressed before others can change [10,110]. Severe sleep disruption may need early intervention because it undermines attention and consolidation [10,110]. Substance use or benzodiazepine dependence may require coordinated management because they can function as safety behaviors or impair learning [34,113]. Acute suicidality, severe depression, psychosis, or unstable trauma-related dissociation may require stabilization before anxiety-focused exposure [10]. Conversely, excessive preparation can become avoidance. The clinician must distinguish necessary sequencing from indefinite postponement of change [10,110].

Combined treatment with pharmacotherapy is often appropriate. SSRIs and SNRIs are evidence-based pharmacological treatments for several anxiety disorders and may reduce symptom burden enough for psychotherapy to proceed [34]. Pharmacotherapy may be particularly useful when anxiety is severe, comorbid depression is present, sleep is disrupted, or physiological arousal prevents engagement [8,34]. The combination should be formulated carefully. Medication can support learning by reducing overwhelming arousal, but it can also become a safety signal if the patient attributes all coping to medication or uses sedatives to avoid exposure [34,113]. Benzodiazepines require particular caution in exposure-based work because of dependence risk, cognitive effects, and potential interference with fear learning in some contexts [34,113]. Medication decisions should be individualized and coordinated with the psychotherapeutic mechanism [10,34].

When pharmacotherapy is used alongside exposure or other learning-based interventions, clinicians should document medication timing, perceived medication effects during therapeutic tasks, and the patient’s attribution of successful coping, because medication that reduces overwhelming arousal may support learning, whereas medication experienced as the reason exposure was tolerable may function as a safety signal.

Measurement-based care should be mechanism-based as well as symptom-based [87,114]. Symptom scales are necessary but insufficient. Clinicians should track avoidance, safety behaviors, exposure generalization, intolerance of uncertainty, attentional focus, sleep, and interpersonal ruptures [87]. If symptoms improve but avoidance remains unchanged, relapse risk may remain high [10,110]. If exposure frequency increases but feared expectancies remain untested, the mechanism has not shifted [110]. If the alliance is polite but the patient withholds shame, relational threat may still block progress [32,112].

To improve clinical implementation, mechanism-based monitoring can be operationalized through brief self-report measures and single-session probes rather than relying only on full research batteries. These markers should be used as formulation and progress-monitoring aids, not as validated treatment-selection thresholds. Table 6 lists candidate clinically feasible markers for each failure mode and distinguishes them from measures that remain primarily research tools.

Supervision and training should also shift from protocol adherence alone to mechanism fidelity [17,55,111]. A therapist may follow a manual but miss the mechanism [17,111]. Assigning exposure without identifying expectancies is formally correct but mechanistically weak [17,111]. Challenging thoughts without addressing reassurance seeking may reinforce worry. Providing relaxation before every exposure may inadvertently teach that anxiety must be reduced before approach [17,111]. Good supervision should ask: What is the maintenance mechanism? What is the therapeutic mechanism? What evidence shows that the mechanism is changing? [17,55,111].

The framework is also relevant for communicating with patients [8]. Patients who have failed previous treatments often arrive demoralized and may interpret non-response as proof that they are untreatable [10,110]. A mechanistic explanation is clinically containing; it allows the clinician to say that previous treatment may not have targeted the right process or may not have done so under the right learning conditions [10]. This is not false reassurance. It is a precise way of preserving hope while maintaining scientific restraint [8]. Clinicians might explain this to patients by saying: This does not mean that you are resistant or untreatable; it means that we need to understand which maintenance process the previous treatment did not sufficiently reach and adjust the next step accordingly.

## 9. Future Directions

Future research should test the proposed taxonomy directly rather than only the broader premise that psychotherapy should be personalized [10,115,116]. The first task is to determine whether the six failure modes can be identified reliably in clinical practice [115,117,118]. This will require brief structured assessment tools that help clinicians distinguish impaired inhibitory learning, cognitive rigidity, stress-related learning impairment, attentional dysregulation, attachment-related barriers, and chronic avoidance dominance after an apparently adequate course of psychotherapy [115,117].

A second priority is to examine whether these failure modes predict differential benefits from specific treatment adaptations [10,116]. For example, patients whose non-response is characterized by persistent safety behaviors and poor expectancy violation should be compared after exposure redesign versus simple exposure dose intensification [10,116]. Patients whose cognitive therapy has become repetitive reassurance or probability disputation should be tested after uncertainty-focused and metacognitive interventions [10,119]. Patients who show shutdown, dissociation, or severe sleep disruption during treatment should be studied in designs that evaluate whether sequencing arousal regulation, sleep intervention, or pharmacological support improves subsequent therapeutic learning [8,10].

The taxonomy also requires better measurement of mechanism-level change [120]. Symptom reduction alone is insufficient, because a patient may report less anxiety while avoidance, reassurance seeking, self-monitoring, or family accommodation remain largely unchanged [120,121]. Future studies should repeatedly assess variables such as feared expectancies, safety behaviors, uncertainty tolerance, attentional focus, sleep disruption, physiological arousal, alliance ruptures, and avoidance contingencies [120,122]. These variables should be measured during treatment, not only before and after it, so that investigators can determine whether mechanism change precedes symptom improvement and whether failure to change a mechanism predicts relapse [120,122].

Adaptive trial designs are particularly suited to this question [115,123]. Rather than treating non-response as an endpoint, trials could use early non-response as a decision point [115,123]. Patients who do not improve after an initial evidence-based intervention could be reclassified according to their dominant failure mode and randomized to either continued standard treatment, increased treatment dose, or mechanism-matched adaptation [116,123]. Such designs would test the clinical value of the taxonomy more directly than conventional trials comparing one branded psychotherapy with another [115,123].

Research should also clarify which failure modes are disorder-specific and which are genuinely transdiagnostic [10]. Impaired inhibitory learning may be especially visible in panic disorder, agoraphobia, social anxiety disorder, and specific phobia, whereas intolerance of uncertainty may be more prominent in generalized anxiety disorder and related presentations [10,119]. Attentional dysregulation may be expressed differently in panic disorder, where interoceptive monitoring is central, than in social anxiety disorder, where self-focused attention and monitoring of social threat are more prominent [121,124]. Attachment-related barriers may cut across diagnoses but may be particularly important when anxiety is embedded in shame, dependency fears, interpersonal trauma, or repeated relational rupture [125].

Future work should also examine implementation [122,126]. A taxonomy that requires extensive specialist assessment may have limited value in ordinary clinical services [122,126]. The field needs concise clinical instruments, supervision models, and measurement routines that can be used in outpatient psychiatry, primary care psychological services, and stepped-care systems [122,126,127]. These tools should help clinicians decide whether to redesign exposure, shift from cognitive content to uncertainty tolerance, address attentional capture, stabilize conditions for learning, repair relational threat, or modify avoidance contingencies outside the consulting room [117,118].

Finally, the taxonomy should be tested against patient-centered outcomes [9,116]. The aim is not only to reduce symptom scores, but to improve functioning, generalization of learning, relapse resilience, and the patient’s capacity to approach feared situations without excessive safety behavior [9,116]. If the proposed failure modes can be identified reliably and linked to better treatment adaptation, psychotherapy non-response may become less of a terminal judgment and more of a clinically useful signal for reformulation [9].

## 10. Limitations of the Framework

This framework has limitations. First, it is a narrative synthesis rather than a systematic review or meta-analysis [10,116]. It integrates evidence across domains but does not provide pooled effect estimates for each proposed failure mode. Second, several mechanisms are better supported than others [10]. Fear extinction, inhibitory learning, avoidance, CBT efficacy, and intolerance of uncertainty have substantial empirical bases [8,10,119]. Attachment-related barriers, mentalization processes, and some stress-learning interactions are clinically persuasive but less directly validated as treatment-matching variables in anxiety disorders [125]. Third, the proposed taxonomy may oversimplify complex clinical presentations [9]. Patients often have multiple interacting mechanisms. A patient may show intolerance of uncertainty, attentional dysregulation, avoidance dominance, and attachment insecurity simultaneously [9]. The framework should therefore be used as a formulation aid, not a rigid classification system. Fourth, biomarkers are not yet clinically decisive [125]. Neuroimaging and cortisol findings are promising but insufficient for routine matching [125]. A maintenance-mechanism-based framework must not be mistaken for an established precision algorithm. Fifth, diagnostic heterogeneity remains a challenge [10,124]. The proposed failure modes are transdiagnostic, but their clinical expression is likely to differ across panic disorder, generalized anxiety disorder, social anxiety disorder, agoraphobia, specific phobia, and separation anxiety presentations [10]. For example, attentional dysregulation may involve interoceptive monitoring in panic disorder, self-focused attention in social anxiety disorder, and threat scanning under uncertainty in generalized anxiety disorder [121,124]. The framework should therefore be used alongside disorder-specific knowledge rather than as a substitute for it [10,124].

A further limitation concerns implementation [122,126]. Mechanism-based formulation requires training, supervision, time, and measurement capacity [122,126]. In many health systems, psychotherapy is delivered under time constraints and with variable access to specialist supervision [126]. A framework that is conceptually attractive but impractical would have limited value [122,126]. Future work should therefore develop brief, reliable, clinically usable tools for identifying dominant failure modes and guiding treatment adaptation [122,126,127].

## 11. Conclusions

Psychotherapy failure in anxiety disorders is clinically heterogeneous. In some cases, it reflects insufficient treatment dose, limited access, poor adherence, comorbidity, therapist factors, or ongoing environmental stressors. In others, particularly after an apparently adequate course of evidence-based psychotherapy, non-response may indicate that the treatment did not sufficiently engage the pathogenic process maintaining anxiety.

The taxonomy proposed in this review offers one way to organize such cases. It distinguishes recurrent patterns of mechanistic non-response: impaired inhibitory learning, cognitive rigidity, stress-related impairment of learning, attentional dysregulation, attachment-related barriers, and chronic avoidance dominance. These categories are not intended as fixed subtypes of anxiety disorders. They are formulation patterns that may help clinicians ask more precise questions after treatment has stalled.

If exposure has failed, the question becomes whether the feared expectancy was actually tested, whether safety behaviors remained active, and whether inhibitory learning generalized beyond the treatment context. If cognitive therapy has failed, the question is whether treatment reduced reassurance seeking and intolerance of uncertainty or merely refined them. If therapy has stalled, the clinician may need to consider whether sleep disruption, hyperarousal, attentional capture, relational threat, or external avoidance contingencies are preventing therapeutic learning.

This reframing has practical clinical value. It supports more precise formulation, more rational sequencing of interventions, and more disciplined integration of psychotherapy with pharmacotherapy and relational work. It also generates empirical questions: whether these failure modes can be identified reliably, whether mechanism-matched adaptation improves outcomes, and whether repeated measurement of mechanism-level change predicts recovery and relapse better than symptom severity alone.

Anxiety disorders remain highly treatable, but non-response should prompt careful reformulation rather than simple repetition of the same intervention. The clinical task is not to decide whether the patient is resistant in a global sense, but to determine what the treatment has not yet reached.

## Figures and Tables

**Table 1:** How the proposed framework differs from adjacent models.

Existing Approach	Guiding Question	Primary Unit of Formulation	Main Contribution	Unresolved Clinical Problem Addressed Here
Disorder-specific CBT protocols	Which evidence-based protocol fits this disorder?	DSM/ICD diagnosis	Provide strong disorder-specific efficacy evidence and scalable treatment procedures	Do not explain why an apparently adequate protocol fails in a specific patient
Process-based therapy	Which modifiable process should be targeted idiographically?	Process of change	Moves psychotherapy beyond protocol packages toward mechanism-level intervention	Less explicitly organized around recurrent patterns of non-response after an adequate therapy trial
Research Domain Criteria (RDoC)	Which transdiagnostic construct explains psychopathology across units of analysis?	Biobehavioral domain, e.g., threat, arousal, cognition	Integrates behavior, circuits, physiology, and self-report	Primarily a research framework rather than a post-treatment clinical decision pathway
Moderator and mediator research	For whom, and through what pathway, does treatment work?	Baseline predictors and within-treatment change variables	Tests differential response and mechanisms statistically	Clinically actionable moderators for anxiety psychotherapy remain inconsistent
Inhibitory-learning exposure models	How can exposure produce more durable fear reduction?	Fear extinction and inhibitory learning	Refines exposure design, expectancy violation, generalization, and relapse prevention	Explains a major exposure-specific failure pathway, but not the broader range of cognitive, attentional, stress-related, relational, and avoidance-dominant failures
Present framework	What did this apparently adequate psychotherapy fail to engage?	Mechanistic failure mode	Links pathogenesis, disrupted therapeutic mechanism, clinical signature, and corrective adaptation	Uses the pattern of non-response to identify the unmodified maintaining process and guide redesign, sequencing, augmentation, or relational/behavioral adaptation

**Table 2:** Mechanistic failure modes in psychotherapy.

Failure Mode	Dominant Maintaining Process	Strength of Evidence	Typical Clinical Signature	Mechanism-Based Treatment Adaptation
Impaired inhibitory learning	Failure to acquire, retrieve, or generalize inhibitory threat learning; persistence of safety behaviors	Strong; supported by translational fear-learning research and clinical exposure literature	Exposure is completed, but expectancy change is limited, gains remain context-bound, or fear rapidly returns	Redesign exposure to maximize expectancy violation, reduce safety behaviors, vary context, and strengthen retrieval/generalization
Cognitive rigidity	Intolerance of uncertainty, perseverative worry, and inflexible threat appraisal	Moderate to strong; robust association with anxiety, with suggestive process-level evidence	Sessions become repetitive debates, reassurance provides only transient relief, and doubt continues to block action	Shift from probability correction to uncertainty tolerance, behavioral experiments, and reduction in reassurance-driven cognitive control
Stress-induced learning impairment	Hyperarousal, sleep disruption, impaired extinction retrieval, and reduced executive control under stress	Moderate; strong experimental basis, but clinical prediction evidence remains mixed	Treatment evokes flooding, shutdown, poor retention, or worsening after sessions	Sequence treatment by stabilizing sleep/arousal, titrating exposure, and considering pharmacological support when clinically indicated
Attentional dysregulation	Persistent threat capture, self-focused attention, or interoceptive monitoring during therapeutic tasks	Moderate; clinically relevant, though experimental findings are heterogeneous	Behavioral participation occurs without corrective learning because attention remains organized around threat monitoring	Strengthen attentional flexibility through external-focus training, mindfulness-based attention regulation, and interoceptive or attentional exercises
Attachment-related barriers	Mistrust, shame, dependency threat, and rupture sensitivity interfering with collaboration and learning	Emerging to moderate; clinically important, with less direct mechanistic validation	Superficial compliance, limited disclosure, withdrawal after perceived criticism, or shame-based disengagement	Strengthen relational safety, address rupture-repair explicitly, and integrate formulation focused on shame, mistrust, and interpersonal threat
Chronic avoidance dominance	Negative reinforcement, habitual avoidance, accommodation, and progressive functional narrowing	Strong clinical and behavioral rationale; individualized assessment remains essential	Homework is repeatedly not implemented, safety behaviors persist, and life becomes organized around avoidance	Use functional analysis, motivational enhancement, values-based activation, reduction in accommodation, and exposure embedded in meaningful goals

**Table 3:** Qualitative strength of evidence supporting proposed mechanistic failure modes.

Failure Mode	Qualitative Evidence Strength	Main Evidentiary Basis	Main Limitation
Impaired inhibitory learning	Strong	Experimental fear-learning literature, inhibitory-learning exposure models, return-of-fear and relapse research, and clinical exposure principles	Individual prediction of exposure failure remains imperfect; relapse may reflect several mechanisms rather than extinction failure alone
Cognitive rigidity/intolerance of uncertainty	Moderate to strong	Meta-analytic evidence linking intolerance of uncertainty with anxiety and worry; CBT, metacognitive, and uncertainty-focused treatment models	Mechanism-specific thresholds for treatment selection and sequencing are not yet well validated
Stress-related learning impairment	Emerging to moderate; primarily translational	Experimental and translational evidence that acute stress, hyperarousal, sleep disruption, and impaired extinction retrieval can compromise learning and regulation; clinical evidence for psychotherapy sequencing remains indirect	Direct clinical trials testing stress-sequenced psychotherapy adaptations remain limited; sequencing recommendations should be treated as formulation hypotheses requiring pilot testing, mechanism-level monitoring, and prospective validation before broad routine implementation
Attentional dysregulation	Moderate	Evidence on threat-related attentional bias, self-focused attention, interoceptive monitoring, and attentional control in anxiety disorders	Task-based attentional-bias findings are heterogeneous and not sufficiently reliable for individual treatment selection
Attachment-related barriers	Emerging to moderate for clinical association; emerging for treatment matching	Adult attachment, social anxiety, alliance rupture, shame, trauma, and interpersonal psychotherapy/process literature; evidence is clinically convergent but mostly indirect for anxiety-specific treatment matching	Direct evidence for attachment-related barriers as anxiety-specific treatment-matching variables remains limited; relational-first adaptations require prospective pilot testing, mechanism-level monitoring, and controlled evaluation before broad routine implementation
Chronic avoidance dominance	Strong behavioral rationale; moderate individualized prediction	Behavioral reinforcement models, safety-behavior literature, family accommodation research, and studies linking avoidance with functional impairment	Avoidance is robust as a maintaining process, but better measurement is needed to predict when it will dominate treatment outcome in a given patient

**Table 4:** Maintenance-mechanism-based treatment matching model.

Dominant Maintaining Process	Therapeutic Mechanism That Failed to Engage	Common Mismatch	Mechanism-Matched Adaptation
Catastrophic interoceptive fear	Inhibitory learning about bodily sensations	Verbal reassurance or generic relaxation	Interoceptive exposure, explicit expectancy violation, and reduction in checking, escape plans, and other safety behaviors
Intolerance of uncertainty	Tolerance of ambiguity and flexible action under uncertainty	Probability-focused cognitive disputation alone	Uncertainty exposure, behavioral experiments, reduction in reassurance, and metacognitive or acceptance-based work
Social-evaluative threat with self-monitoring	Processing of external social information and corrective interpersonal feedback	Social exposure while internally scanning for anxiety signs or disapproval	External-focus training, video feedback, attentional flexibility, and behavioral experiments targeting specific social predictions
Chronic hyperarousal and sleep disruption	Encoding, consolidation, and retrieval of corrective learning	Premature high-intensity exposure without adequate learning conditions	Sequenced sleep/arousal stabilization, carefully titrated exposure, and pharmacotherapy when clinically indicated
Attachment insecurity and shame	Use of the therapeutic relationship as a secure base for learning	Manualized technique without rupture repair or attention to relational threat	Relational formulation, explicit rupture repair, validation before challenge, and work with shame, mistrust, and dependency threat
Family- or partner-accommodated avoidance	Reinforcement shift from avoidance to approach	Homework assignments without changing external contingencies	Functional analysis, reduction in accommodation, values-based exposure, contingency planning, and systemic involvement when relevant

**Table 5:** Disorder-specific expressions of mechanistic non-response in anxiety disorders.

Disorder/Clinical Presentation	Common Dominant Maintaining Mechanism	Most Likely Failure Mode(s)	Typical Clinical Signature of Non-Response	Corrective Treatment Implication
Panic disorder	Catastrophic interpretation of bodily sensations, interoceptive avoidance, and anxiety sensitivity	Impaired inhibitory learning; attentional dysregulation	Cognitive understanding is present, but bodily catastrophe remains untested and avoidance of arousal persists	Use interoceptive exposure, explicitly test feared bodily predictions, and reduce checking, reassurance, and medication-based safety behaviors
Agoraphobia	Context-bound fear of being trapped, helpless, or unable to escape; reliance on companions or safety zones	Impaired inhibitory learning; chronic avoidance dominance	Exposure is completed only under protected conditions, and gains fail to generalize across settings	Use graded but variable in vivo exposure, systematically fade safety signals and companions, and extend exposure across contexts and uncertainty levels
Social anxiety disorder	Fear of negative evaluation, shame, self-focused attention, and post-event processing	Attentional dysregulation; attachment-related barriers; impaired inhibitory learning	Social tasks are completed, but persistent self-monitoring and negative post-event interpretation prevent corrective learning	Use external-focus training, video feedback, and behavioral experiments targeting social predictions; address shame and relational threat when prominent
Generalized anxiety disorder	Intolerance of uncertainty, worry as attempted control, reassurance seeking, and over-preparation	Cognitive rigidity; chronic avoidance dominance	Cognitive work becomes repetitive debate, and unresolved doubt continues to block action	Shift toward uncertainty exposure, reduce reassurance and checking, and use behavioral experiments targeting tolerance of uncertainty rather than probability alone
Specific phobia	Circumscribed conditioned fear and avoidance of a specific object or situation	Impaired inhibitory learning	Exposure is brief, predictable, safety behavior-dependent, or context-bound, with rapid return of fear	Design exposure around expectancy violation, variability, and generalization; reduce safety behaviors; use applied tension when blood-injection-injury physiology is relevant
Separation anxiety/dependency-related anxiety in adults	Fear of separation, abandonment, loss of attachment figure, or inability to cope alone	Attachment-related barriers; chronic avoidance dominance	Fear can be discussed cognitively, but reliance on proximity, repeated contact, checking, or reassurance persists	Use graded separation experiments, reduce reassurance cycles, and address attachment expectations, autonomy, and relational safety
Mixed anxiety with trauma or chronic stress load	Hyperarousal, sleep disruption, threat generalization, and impaired consolidation of new learning	Stress-induced learning impairment; attentional dysregulation	Treatment evokes flooding, shutdown, dissociation, poor retention, or worsening sleep rather than durable learning	Sequence sleep and arousal stabilization, titrate exposure carefully, use grounding to facilitate learning rather than avoidance, and consider pharmacotherapy when indicated

**Table 6:** Candidate brief markers for mechanism-based monitoring of psychotherapy non-response.

Failure Mode	Candidate Clinical Markers or Single-Session Probes	Clinical Use Status
Impaired inhibitory learning	Exposure learning record documenting target expectancy, pre- and post-exposure belief rating, actual outcome, safety behaviors used, distress trajectory, and generalization outside the session. Single-session probes: Which feared prediction was tested?, What expectancy changed?, Which safety behaviors remained active?, Was the learning retrieved in daily-life contexts?	Clinically feasible for process monitoring. Laboratory extinction paradigms, psychophysiology, and neuroimaging remain research tools and are not ready for routine individual treatment selection.
Cognitive rigidity/intolerance of uncertainty	Brief intolerance-of-uncertainty rating or short IU measure; frequency of reassurance seeking, checking, over-preparation, or decision avoidance since the last session; 0–10 rating of ability to act without certainty. Single-session probe: What did worry, checking, or analysis allow the patient to avoid doing?	Clinically feasible as a formulation and progress-monitoring marker. Validated thresholds for selecting or sequencing specific interventions are not yet established.
Stress-related learning impairment	Sleep diary or Insomnia Severity Index; sleep duration and perceived sleep quality before and after therapeutic tasks; 0–10 ratings of hyperarousal, dissociation, shutdown, or flooding during exposure; sedative medication, alcohol, or substance use before learning tasks; recall of session learning at the next visit.	Sleep, arousal, dissociation, and medication-use monitoring are clinically feasible. Cortisol, polysomnography, fMRI, and experimental stress-learning paradigms remain research or specialist tools.
Attentional dysregulation	During exposure, brief attention-allocation rating: self-monitoring, bodily scanning, external task focus, threat scanning, and corrective cue processing. Additional probes include recall of neutral or disconfirmatory social information and post-event processing after social tasks.	Clinically feasible as session-level process monitoring. Dot-probe tasks, eye-tracking, and experimental attentional-bias indices are not sufficiently reliable for routine individual treatment selection.
Attachment-related barriers	Brief alliance or session-feedback measure; rupture probe: Did anything in the session feel critical, unsafe, coercive, dismissive, or shaming?; 0–10 ratings of shame, mistrust, or fear of disappointing the therapist; disclosure probe: What felt difficult or unsafe to say today?	Clinically feasible for monitoring engagement, rupture risk, and supervision needs. Formal attachment interviews and complex attachment classifications remain specialist or research assessments in this context.
Chronic avoidance dominance	Homework implementation rate; avoided-situation count; safety-behavior log; functional analysis of short-term reinforcement from avoidance; family or partner accommodation checklist; Work and Social Adjustment Scale when functional impairment is central.	Clinically feasible for routine monitoring. Passive digital phenotyping, actigraphy-based avoidance detection, and algorithmic prediction remain research tools.

## Data Availability

No new data were created or analyzed in this study. Data sharing is not applicable to this article.

## Supplementary Materials

The following supporting information can be downloaded at: https://www.mdpi.com/article/10.3390/jcm15114223/s1, Supplementary Table S1: SANRA self-check for the structured narrative review; Table S2: Search strategy and evidence mapping; Table S3: Clinician Rapid Guide: Mechanistic Non-Response After Psychotherapy for Anxiety Disorders.

## Abbreviations

The following abbreviations are used in this manuscript:

CBT	Cognitive-behavioral therapy
DSM-5-TR	Diagnostic and Statistical Manual of Mental Disorders, Fifth Edition, Text Revision
fMRI	Functional magnetic resonance imaging
HPA	Hypothalamic–pituitary–adrenal
ICD-11	International Classification of Diseases, 11th Revision
RDoC	Research Domain Criteria
SANRA	Scale for the Assessment of Narrative Review Articles
SNRI	Serotonin-norepinephrine reuptake inhibitor
SSRI	Selective serotonin reuptake inhibitor

## References

[1] Xiong P., Liu M., Liu B., Hall B.J. (2022). Trends in the incidence and DALYs of anxiety disorders at the global, regional, and national levels: Estimates from the Global Burden of Disease Study 2019. J. Affect. Disord..

[2] Wu Y., Li X., Ji X., Ren W., Zhu Y., Chen Z., Du X. (2025). Trends in the epidemiology of anxiety disorders from 1990 to 2021: A global, regional, and national analysis with a focus on the sociodemographic index. J. Affect. Disord..

[3] Zhou J., Li S., Song Y., Ying J., Luo Z., Shan S., Zhou L., Zha J., Wang X., Song P. (2025). Global, Regional, and National Trends in the Burden of Anxiety Disorders From 1992 to 2021: An Age-Period-Cohort Analysis Based on the Global Burden of Disease Study 2021. Depress. Anxiety.

[4] (2021). COVID-19 Mental Disorders Collaborators. Global prevalence and burden of depressive and anxiety disorders in 204 countries and territories in 2020 due to the COVID-19 pandemic. Lancet.

[5] James A.C., Reardon T., Soler A., James G., Creswell C. (2020). Cognitive behavioural therapy for anxiety disorders in children and adolescents. Cochrane Database Syst. Rev..

[6] van Dis E.A.M., van Veen S.C., Hagenaars M.A., Batelaan N.M., Bockting C.L.H., van den Heuvel R.M., Cuijpers P., Engelhard I.M. (2020). Long-term Outcomes of Cognitive Behavioral Therapy for Anxiety-Related Disorders: A Systematic Review and Meta-analysis. JAMA Psychiatry.

[7] Shepardson R.L., Khan J.S., Buckheit K.A., Funderburk J.S. (2026). Treatment of Anxiety for Adults in Primary Care Settings: A Review. JAMA Intern. Med..

[8] Penninx B.W., Pine D.S., Holmes E.A., Reif A. (2021). Anxiety disorders. Lancet.

[9] Taylor S., Abramowitz J.S., McKay D. (2012). Non-adherence and non-response in the treatment of anxiety disorders. J. Anxiety Disord..

[10] Schiele M.A., Fagan H.A., Baldwin D.S., Domschke K. (2025). Integrative Systematic Review on Pharmacological, Psychotherapeutic, and Neurostimulatory Treatment Options in Treatment-Resistant Anxiety Disorders. Psychother. Psychosom..

[11] Roy-Byrne P. (2015). Treatment-refractory anxiety; definition, risk factors, and treatment challenges. Dialogues Clin. Neurosci..

[12] Craske M.G., Treanor M., Zbozinek T.D., Vervliet B. (2022). Optimizing exposure therapy with an inhibitory retrieval approach and the OptEx Nexus. Behav. Res. Ther..

[13] Craske M.G., Sandman C.F., Stein M.B. (2022). How can neurobiology of fear extinction inform treatment?. Neurosci. Biobehav. Rev..

[14] Craske M.G., Treanor M., Conway C.C., Zbozinek T., Vervliet B. (2014). Maximizing exposure therapy: An inhibitory learning approach. Behav. Res. Ther..

[15] Levy K.N., Kivity Y., Johnson B.N., Gooch C.V. (2018). Adult attachment as a predictor and moderator of psychotherapy outcome: A meta-analysis. J. Clin. Psychol..

[16] Philippot P., Bouvard M., Baeyens C., Dethier V. (2019). Case conceptualization from a process-based and modular perspective: Rationale and application to mood and anxiety disorders. Clin. Psychol. Psychother..

[17] Haug T., Nordgreen T., Öst L.G., Tangen T., Kvale G., Hovland O.J., Heiervang E.R., Havik O.E. (2016). Working alliance and competence as predictors of outcome in cognitive behavioral therapy for social anxiety and panic disorder in adults. Behav. Res. Ther..

[18] Marin M.F., Hammoud M.Z., Klumpp H., Simon N.M., Milad M.R. (2020). Multimodal Categorical and Dimensional Approaches to Understanding Threat Conditioning and Its Extinction in Individuals with Anxiety Disorders. JAMA Psychiatry.

[19] Hayes S.C., Hofmann S.G., Ciarrochi J. (2020). A process-based approach to psychological diagnosis and treatment: The conceptual and treatment utility of an extended evolutionary meta model. Clin. Psychol. Rev..

[20] Stangier U., Kohl V., Görg N., Sendig L., Hufschmidt B., Bonarius D., Nemani A., Ebert M., Hofmann S.G. (2024). Process-based therapy vs. routine-CBT for difficult-to-treat mood and anxiety disorders: Study protocol for a randomized controlled trial. Trials.

[21] Schenker M.T., Ney L.J., Miller L.N., Felmingham K.L., Nicholas C.L., Jordan A.S. (2021). Sleep and fear conditioning, extinction learning and extinction recall: A systematic review and meta-analysis of polysomnographic findings. Sleep. Med. Rev..

[22] Bottary R., Straus L.D., Pace-Schott E.F. (2023). The Impact of Sleep on Fear Extinction. Curr. Top. Behav. Neurosci..

[23] Wilson E.J., Abbott M.J., Norton A.R. (2023). The impact of psychological treatment on intolerance of uncertainty in generalized anxiety disorder: A systematic review and meta-analysis. J. Anxiety Disord..

[24] Morriss J. (2025). Psychological mechanisms underpinning change in intolerance of uncertainty across anxiety-related disorders: New insights for translational research. Neurosci. Biobehav. Rev..

[25] Seo J., Yuksel C., Oliver K.I., Daffre C., Song H., Lasko N.B., McCoy E.R.S., Milad M.R., Min B.K., Pace-Schott E.F. (2026). Local and network neural activations and their associations with sleep parameters during threat conditioning and extinction in persons with generalized anxiety disorder with and without insomnia disorder. Psychiatry Res. Neuroimaging.

[26] Seo J., Oliver K.I., Daffre C., Moore K.N., Gazecki S., Lasko N.B., Milad M.R., Pace-Schott E.F. (2022). Associations of sleep measures with neural activations accompanying fear conditioning and extinction learning and memory in trauma-exposed individuals. Sleep.

[27] Sauer-Zavala S., Boswell J.F., Bentley K.H., Thompson-Hollands J., Farchione T.J., Barlow D.H. (2018). Expectancies, working alliance, and outcome in transdiagnostic and single diagnosis treatment for anxiety disorders: An investigation of mediation. Cogn. Ther. Res..

[28] Craske M.G., Hermans D., Vervliet B. (2018). State-of-the-art and future directions for extinction as a translational model for fear and anxiety. Philos. Trans. R. Soc. Lond. B Biol. Sci..

[29] Wen Z., Seo J., Pace-Schott E.F., Milad M.R. (2022). Abnormal dynamic functional connectivity during fear extinction learning in PTSD and anxiety disorders. Mol. Psychiatry.

[30] Marin M.F., Zsido R.G., Song H., Lasko N.B., Killgore W.D.S., Rauch S.L., Simon N.M., Milad M.R. (2017). Skin Conductance Responses and Neural Activations During Fear Conditioning and Extinction Recall Across Anxiety Disorders. JAMA Psychiatry.

[31] Dugas M.J., Sexton K.A., Hebert E.A., Bouchard S., Gouin J.P., Shafran R. (2022). Behavioral Experiments for Intolerance of Uncertainty: A Randomized Clinical Trial for Adults with Generalized Anxiety Disorder. Behav. Ther..

[32] Luong H.K., Drummond S.P.A., Norton P.J. (2020). Elements of the therapeutic relationship in CBT for anxiety disorders: A systematic review. J. Anxiety Disord..

[33] Baethge C., Goldbeck-Wood S., Mertens S. (2019). SANRA—A scale for the quality assessment of narrative review articles. Res. Integr. Peer Rev..

[34] Szuhany K.L., Simon N.M. (2022). Anxiety Disorders: A Review. JAMA.

[35] Bokma W.A., Wetzer G.A.A.M., Gehrels J.B., Penninx B.W.J.H., Batelaan N.M., van Balkom A.L.J.M. (2019). Aligning the many definitions of treatment resistance in anxiety disorders: A systematic review. Depress. Anxiety.

[36] Blakey S.M., Abramowitz J.S. (2016). The effects of safety behaviors during exposure therapy for anxiety: Critical analysis from an inhibitory learning perspective. Clin. Psychol. Rev..

[37] Im Brahm C., Heinig I., Goerigk S., Arolt V., Bartnick C., Dannlowski U., Deckert J., Domschke K., Fydrich T., Hamm A.O. (2026). Utilization of different types of safety behavior during exposure-based CBT for anxiety disorders and its correlates. Cogn. Behav. Ther..

[38] Sharpe L., Todd J., Scott A., Gatzounis R., Menzies R.E., Meulders A. (2022). Safety behaviours or safety precautions? The role of subtle avoidance in anxiety disorders in the context of chronic physical illness. Clin. Psychol. Rev..

[39] Kirk A., Meyer J.M., Whisman M.A., Deacon B.J., Arch J.J. (2019). Safety behaviors, experiential avoidance, and anxiety: A path analysis approach. J. Anxiety Disord..

[40] Lorimer B., Kellett S., Nye A., Delgadillo J. (2021). Predictors of relapse and recurrence following cognitive behavioural therapy for anxiety-related disorders: A systematic review. Cogn. Behav. Ther..

[41] Levy H.C., O’Bryan E.M., Tolin D.F. (2021). A meta-analysis of relapse rates in cognitive-behavioral therapy for anxiety disorders. J. Anxiety Disord..

[42] Hendriks G.J., Janssen N., Robertson L., van Balkom A.J., van Zelst W.H., Wolfe S., Oude Voshaar R.C., Uphoff E. (2024). Cognitive behavioural therapy and third-wave approaches for anxiety and related disorders in older people. Cochrane Database Syst. Rev..

[43] Weisman J.S., Rodebaugh T.L. (2018). Exposure therapy augmentation: A review and extension of techniques informed by an inhibitory learning approach. Clin. Psychol. Rev..

[44] Stemerding L.E., van Ast V.A., Kindt M. (2023). Manipulating expectancy violations to strengthen the efficacy of human fear extinction. Behav. Res. Ther..

[45] Goodman F.R., Peckham A.D., Kneeland E.T., Choate A.M., Daniel K.E., Beard C., Björgvinsson T. (2023). How does emotion regulation change during psychotherapy? A daily diary study of adults in a transdiagnostic partial hospitalization program. J. Consult. Clin. Psychol..

[46] O’Toole M.S., Renna M.E., Mennin D.S., Fresco D.M. (2019). Changes in Decentering and Reappraisal Temporally Precede Symptom Reduction During Emotion Regulation Therapy for Generalized Anxiety Disorder with and without Co-Occurring Depression. Behav. Ther..

[47] Miller M.L., McGuire J.F. (2023). Targeting intolerance of uncertainty in treatment: A meta-analysis of therapeutic effects, treatment moderators, and underlying mechanisms. J. Affect. Disord..

[48] Mogg K., Bradley B.P. (2016). Anxiety and attention to threat: Cognitive mechanisms and treatment with attention bias modification. Behav. Res. Ther..

[49] Fodor L.A., Georgescu R., Cuijpers P., Szamoskozi Ş., David D., Furukawa T.A., Cristea I.A. (2020). Efficacy of cognitive bias modification interventions in anxiety and depressive disorders: A systematic review and network meta-analysis. Lancet Psychiatry.

[50] Mogg K., Bradley B.P. (2018). Anxiety and Threat-Related Attention: Cognitive-Motivational Framework and Treatment. Trends Cogn. Sci..

[51] Fergus T.A., Wheless N.E., Wright L.C. (2014). The attention training technique, self-focused attention, and anxiety: A laboratory-based component study. Behav. Res. Ther..

[52] Klumpp H., Davey D., Langenecker S.A. (2026). Neural predictors of treatment outcome through emotion regulation in internalizing disorders: A narrative review. Transl. Psychiatry.

[53] Grecucci A., Messina I., Amodeo L., Lapomarda G., Crescentini C., Dadomo H., Panzeri M., Theuninck A., Frederickson J. (2020). A Dual Route Model for Regulating Emotions: Comparing Models, Techniques and Biological Mechanisms. Front. Psychol..

[54] Lange J., Goerigk S., Nowak K., Rosner R., Erhardt A. (2021). Attachment style change and working alliance in panic disorder patients treated with cognitive behavioral therapy. Psychotherapy.

[55] Bjaastad J.F., Gjestad R., Fjermestad K., Öst L.G., Haugland B.S.M., Kodal A., Heiervang E.R., Wergeland G.J. (2023). Adherence, Competence, and Alliance as Predictors of Long-term Outcomes of Cognitive Behavioral Therapy for Youth Anxiety Disorders. Res. Child. Adolesc. Psychopathol..

[56] Jacobsen C.F., Falkenström F., Castonguay L., Nielsen J., Lunn S., Lauritzen L., Poulsen S. (2024). The relationship between attachment needs, earned secure therapeutic attachment and outcome in adult psychotherapy. J. Consult. Clin. Psychol..

[57] Hang Y., Xu L., Wang C., Zhang G., Zhang N. (2021). Can attention bias modification augment the effect of CBT for anxiety disorders? A systematic review and meta-analysis. Psychiatry Res..

[58] Nielsen S.K.K., Hageman I., Petersen A., Daniel S.I.F., Lau M., Winding C., Wolitzky-Taylor K.B., Steele H., Vangkilde S. (2019). Do emotion regulation, attentional control, and attachment style predict response to cognitive behavioral therapy for anxiety disorders?—An investigation in clinical settings. Psychother. Res..

[59] Taylor P.J., Rietzschel J., Danquah A., Berry K. (2015). The role of attachment style, attachment to therapist, and working alliance in response to psychological therapy. Psychol. Psychother..

[60] Kausche F.M., Carsten H.P., Sobania K.M., Riesel A. (2025). Fear and safety learning in anxiety- and stress-related disorders: An updated meta-analysis. Neurosci. Biobehav. Rev..

[61] Zheng A., Yu D., Meng X., Gong S., Ye J., Zhou H., Chen M., An S., Ma J., Li C. (2026). Fear extinction induces maladaptive generalization via noradrenergic and GABAergic systems. Proc. Natl. Acad. Sci. USA.

[62] Katz D., Rector N.A., Laposa J.M. (2017). The interaction of distress tolerance and intolerance of uncertainty in the prediction of symptom reduction across CBT for social anxiety disorder. Cogn. Behav. Ther..

[63] Laposa J.M., Katz D.E., Lisi D.M., Hawley L.L., Quigley L., Rector N.A. (2022). Longitudinal changes in intolerance of uncertainty and worry severity during CBT for generalized anxiety disorder. J. Anxiety Disord..

[64] Bayer H., Binette A.N., Sweck S.O., Juliano V.A.L., Plas S.L., Ferst L.M., Hassell J.E., Mourão F.A.G., Maren S. (2026). Locus coeruleus-amygdala circuit disrupts prefrontal control to impair fear extinction. Proc. Natl. Acad. Sci. USA.

[65] Merz C.J., Wolf O.T. (2022). How stress hormones shape memories of fear and anxiety in humans. Neurosci. Biobehav. Rev..

[66] Bottary R., Seo J., Daffre C., Gazecki S., Moore K.N., Kopotiyenko K., Dominguez J.P., Gannon K., Lasko N.B., Roth B. (2020). Fear extinction memory is negatively associated with REM sleep in insomnia disorder. Sleep.

[67] Pace-Schott E.F., Germain A., Milad M.R. (2015). Effects of sleep on memory for conditioned fear and fear extinction. Psychol. Bull..

[68] Adamis A.M., Walske S., Olatunji B.O. (2025). Attention mechanisms of social anxiety in daily life: Unique effects of negative self-focused attention on post-event processing. Behav. Res. Ther..

[69] Iannazzi E.M., Grennan G., Zhao Y., Chang K., Feusner J.D., Wilhelm S., Manoach D.S., Fang A. (2025). Task-based neural correlates of self-focused attention associated with cognitive behavioral therapy response. Biol. Psychol..

[70] Lin M., Wen X., Qian M., He D., Zlomuzica A. (2021). Self-focused attention vs. negative attentional bias during public speech task in socially anxious individuals. Behav. Res. Ther..

[71] Newman M.G., Castonguay L.G., Jacobson N.C., Moore G.A. (2015). Adult attachment as a moderator of treatment outcome for generalized anxiety disorder: Comparison between cognitive-behavioral therapy (CBT) plus supportive listening and CBT plus interpersonal and emotional processing therapy. J. Consult. Clin. Psychol..

[72] Adams G.C., McWilliams L.A., Wrath A.J., Adams S., Souza D. (2017). Relationships between patients’ attachment characteristics and views and use of psychiatric treatment. Psychiatry Res..

[73] Altmann U., Nodop S., Dinger U., Ehrenthal J.C., Schauenburg H., Dymel W., Willutzki U., Strauss B.M. (2021). Differential effects of adult attachment in cognitive-behavioural and psychodynamic therapy in social anxiety disorder: A comparison between a self-rating and an observer rating. Clin. Psychol. Psychother..

[74] Levy K.N., Ellison W.D., Scott L.N., Bernecker S.L. (2011). Attachment style. J. Clin. Psychol..

[75] Coyne A.E., Constantino M.J., Westra H.A., Antony M.M. (2019). Interpersonal change as a mediator of the within- and between-patient alliance-outcome association in two treatments for generalized anxiety disorder. J. Consult. Clin. Psychol..

[76] Kendall P.C., Norris L.A., Rabner J.C., Crane M.E., Rifkin L.S. (2020). Intolerance of Uncertainty and Parental Accommodation: Promising Targets for Personalized Intervention for Youth Anxiety. Curr. Psychiatry Rep..

[77] Henry A.L., Miller C.B., Emsley R., Sheaves B., Freeman D., Luik A.I., Espie C.A. (2023). Does treating insomnia with digital cognitive behavioural therapy (Sleepio) mediate improvements in anxiety for those with insomnia and comorbid anxiety? An analysis using individual participant data from two large randomised controlled trials. J. Affect. Disord..

[78] Shalom J.G., Prihar A., Strauss A.Y., Huppert J.D., Andersson G., Aderka I.M. (2025). The Relationship Between the Therapeutic Alliance and Social Anxiety Symptoms Along the Course of Internet-Delivered Cognitive Behavioral Treatment. Behav. Ther..

[79] Hilleke M., Lang T., Helbig-Lang S., Alpers G.W., Arolt V., Deckert J., Fydrich T., Hamm A.O., Kircher T., Richter J. (2025). How Do Patients’ Fear Prediction and Fear Experience Impact Exposure-Based Therapy for Panic Disorder with Agoraphobia? A Comprehensive Analysis of Fear Prediction. Depress. Anxiety.

[80] Thaon de Saint André S., Heinig I., Arolt V., Bartnick C., Dannlowski U., Deckert J., Domschke K., Fydrich T., Goerigk S., Hamm A.O. (2025). Same same but different: Threat expectancy change and fear reduction as readouts of exposure rationales are only weakly associated and contribute differentially to treatment outcome in anxiety disorders. Behav. Res. Ther..

[81] Zbozinek T.D., Rosenberg B.M., Treanor M., Craske M.G. (2026). Expectancy updating predicts anxiety symptom reduction from exposure therapy: Predictive analyses from a randomized clinical trial. Behav. Res. Ther..

[82] Xue Y., Wang W.D., Liu Y.J., Wang J., Walters A.S. (2025). Sleep disturbances in generalized anxiety Disorder: The central role of insomnia. Sleep Med..

[83] Strauß B., Altmann U., Manes S., Tholl A., Koranyi S., Nolte T., Beutel M.E., Wiltink J., Herpertz S., Hiller W. (2018). Changes of attachment characteristics during psychotherapy of patients with social anxiety disorder: Results from the SOPHO-Net trial. PLoS ONE.

[84] Salkovskis P.M., Hackmann A., Wells A., Gelder M.G., Clark D.M. (2007). Belief disconfirmation versus habituation approaches to situational exposure in panic disorder with agoraphobia: A pilot study. Behav. Res. Ther..

[85] Krzikalla C., Morina N., Andor T., Nohr L., Buhlmann U. (2023). Psychological interventions for generalized anxiety disorder: Effects and predictors in a naturalistic outpatient setting. PLoS ONE.

[86] Kivity Y., Strauss A.Y., Elizur J., Weiss M., Cohen L., Huppert J.D. (2021). The alliance mediates outcome in cognitive-behavioral therapy for social anxiety disorder, but not in attention bias modification. Psychother. Res..

[87] Lewis C.C., Boyd M., Puspitasari A., Navarro E., Howard J., Kassab H., Hoffman M., Scott K., Lyon A., Douglas S. (2019). Implementing Measurement-Based Care in Behavioral Health: A Review. JAMA Psychiatry.

[88] Kendrick T., El-Gohary M., Stuart B., Gilbody S., Churchill R., Aiken L., Bhattacharya A., Gimson A., Brütt A.L., de Jong K. (2016). Routine use of patient reported outcome measures (PROMs) for improving treatment of common mental health disorders in adults. Cochrane Database Syst. Rev..

[89] Ridout K.K., Vanderlip E., Carlo A.D., Kadriu B., Livesey C., Torous J., Alter C. (2025). Considerations for Implementation of Measurement-Based Care: Focus on Solo and Small-Group Practitioners. Psychiatr. Serv..

[90] Dutcher C.D., Dowd S.M., Zalta A.K., Taylor D.J., Rosenfield D., Perrone A., Otto M.W., Pollack M.H., Hofmann S.G., Smits J.A.J. (2021). Sleep quality and outcome of exposure therapy in adults with social anxiety disorder. Depress. Anxiety.

[91] Fadardi J.S., Memarian S., Parkinson J., Cox W.M., Stacy A.W. (2023). Scary in the eye of the beholder: Attentional bias and attentional retraining for social anxiety. J. Psychiatr. Res..

[92] Constantino M.J., Westra H.A., Antony M.M., Coyne A.E. (2019). Specific and common processes as mediators of the long-term effects of cognitive-behavioral therapy integrated with motivational interviewing for generalized anxiety disorder. Psychother. Res..

[93] Kessler R.C., Luedtke A. (2021). Pragmatic Precision Psychiatry—A New Direction for Optimizing Treatment Selection. JAMA Psychiatry.

[94] Perna G., Alciati A., Sangiorgio E., Caldirola D., Nemeroff C.B. (2020). Personalized Clinical Approaches to Anxiety Disorders. Adv. Exp. Med. Biol..

[95] Lueken U., Zierhut K.C., Hahn T., Straube B., Kircher T., Reif A., Richter J., Hamm A., Wittchen H.U., Domschke K. (2016). Neurobiological markers predicting treatment response in anxiety disorders: A systematic review and implications for clinical application. Neurosci. Biobehav. Rev..

[96] Brehl A.K., Kohn N., Schene A.H., Fernández G. (2020). A mechanistic model for individualised treatment of anxiety disorders based on predictive neural biomarkers. Psychol. Med..

[97] Picó-Pérez M., Fullana M.A., Albajes-Eizagirre A., Vega D., Marco-Pallarés J., Vilar A., Chamorro J., Felmingham K.L., Harrison B.J., Radua J. (2023). Neural predictors of cognitive-behavior therapy outcome in anxiety-related disorders: A meta-analysis of task-based fMRI studies. Psychol. Med..

[98] Schrammen E., Roesmann K., Rosenbaum D., Redlich R., Harenbrock J., Dannlowski U., Leehr E.J. (2022). Functional neural changes associated with psychotherapy in anxiety disorders—A meta-analysis of longitudinal fMRI studies. Neurosci. Biobehav. Rev..

[99] Santos V.A., Carvalho D.D., Van Ameringen M., Nardi A.E., Freire R.C. (2019). Neuroimaging findings as predictors of treatment outcome of psychotherapy in anxiety disorders. Prog. Neuropsychopharmacol. Biol. Psychiatry.

[100] Fischer S., Cleare A.J. (2017). Cortisol as a predictor of psychological therapy response in anxiety disorders-Systematic review and meta-analysis. J. Anxiety Disord..

[101] Schmalbach I., Witthöft M., Strauß B., Joraschky P., Petrowski K. (2024). The predictive value of cortisol in psychodynamic psychotherapy for social anxiety disorder: Extended results of the SOPHONET-Study. Transl. Psychiatry.

[102] Fischer S., King S., Papadopoulos A., Hotopf M., Young A.H., Cleare A.J. (2018). Hair cortisol and childhood trauma predict psychological therapy response in depression and anxiety disorders. Acta Psychiatr. Scand..

[103] Masdrakis V.G., Legaki E.M., Papageorgiou C., Markianos M. (2021). Stress Hormones as Predictors of Response to Cognitive Behavior Therapy in Panic Disorder. Neuropsychobiology.

[104] Tyson G., Ferreira V., Shoja-Assadi P., Hirsch C.R. (2025). Testing associations between negative interpretation inflexibility, anxiety symptoms and intolerance of uncertainty. Behav. Res. Ther..

[105] Beard J.I.L., Delgadillo J. (2019). Early response to psychological therapy as a predictor of depression and anxiety treatment outcomes: A systematic review and meta-analysis. Depress. Anxiety.

[106] Vîslă A., Probst G.H., Flückiger C. (2023). Symptom severity in daily life, early response and posttreatment changes in anxiety and depressive symptoms in generalized anxiety disorder. Clin. Psychol. Psychother..

[107] Bufano P., Laurino M., Said S., Tognetti A., Menicucci D. (2023). Digital Phenotyping for Monitoring Mental Disorders: Systematic Review. J. Med. Internet Res..

[108] Jacobson N.C., Bhattacharya S. (2022). Digital biomarkers of anxiety disorder symptom changes: Personalized deep learning models using smartphone sensors accurately predict anxiety symptoms from ecological momentary assessments. Behav. Res. Ther..

[109] Lutz W., Vehlen A., Schwartz B. (2024). Data-informed psychological therapy, measurement-based care, and precision mental health. J. Consult. Clin. Psychol..

[110] Zbozinek T.D., Khalsa S.S., Craske M.G. (2026). New and emerging treatments for anxiety disorders. BMJ.

[111] Wade T.D., Waller G. (2025). Ten generic competences to improve outcomes of cognitive behaviour therapy: Evidence, postulated processes, and clinical implications. Behav. Res. Ther..

[112] Eubanks C.F., Muran J.C., Safran J.D. (2018). Alliance rupture repair: A meta-analysis. Psychotherapy.

[113] Melani M.S., Paiva J.M., Silva M.C., Mendlowicz M.V., Figueira I., Marques-Portella C., Luz M.P., Ventura P.R., Berger W. (2020). Absence of definitive scientific evidence that benzodiazepines could hinder the efficacy of exposure-based interventions in adults with anxiety or posttraumatic stress disorders: A systematic review of randomized clinical trials. Depress. Anxiety.

[114] Lutz W., Schwartz B., Delgadillo J. (2022). Measurement-Based and Data-Informed Psychological Therapy. Annu. Rev. Clin. Psychol..

[115] Langhammer T., Unterfeld C., Blankenburg F., Erk S., Fehm L., Haynes J.D., Heinzel S., Hilbert K., Jacobi F., Kathmann N. (2025). Design and methods of the research unit 5187 PREACT (towards precision psychotherapy for non-respondent patients: From signatures to predictions to clinical utility)—A study protocol for a multicentre observational study in outpatient clinics. BMJ Open.

[116] Gloster A.T., Rinner M.T.B., Ioannou M., Villanueva J., Block V.J., Ferrari G., Benoy C., Bader K., Karekla M. (2020). Treating treatment non-responders: A meta-analysis of randomized controlled psychotherapy trials. Clin. Psychol. Rev..

[117] Schilling V.N.L.S., Zimmermann D., Rubel J.A., Boyle K.S., Lutz W. (2021). Why do patients go off track? Examining potential influencing factors for being at risk of psychotherapy treatment failure. Qual. Life Res..

[118] Williams E., Christensen H., Aguirre E., Wheatley J., Corfe A., Thomson J., Delgadillo J. (2026). Why are some cases not on track? An investigation of common obstacles and solutions during feedback-informed psychological therapy. J. Consult. Clin. Psychol..

[119] Krzikalla C., Buhlmann U., Schug J., Kopei I., Gerlach A.L., Doebler P., Morina N., Andor T. (2024). Worry Postponement From the Metacognitive Perspective: A Randomized Waitlist-Controlled Trial. Clin. Psychol. Eur..

[120] Sønderland N.M., Solbakken O.A., Eilertsen D.E., Nordmo M., Monsen J.T. (2024). Emotional changes and outcomes in psychotherapy: A systematic review and meta-analysis. J. Consult. Clin. Psychol..

[121] Asnaani A., Tyler J., McCann J., Brown L., Zang Y. (2020). Anxiety sensitivity and emotion regulation as mechanisms of successful CBT outcome for anxiety-related disorders in a naturalistic treatment setting. J. Affect. Disord..

[122] Mink F., Lutz W., Hehlmann M.I. (2025). Ecological Momentary Assessment in psychotherapy research: A systematic review. Clin. Psychol. Rev..

[123] Peterson B.S., West A.E., Weisz J.R., Mack W.J., Kipke M.D., Findling R.L., Mittman B.S., Bansal R., Piantadosi S., Takata G. (2021). A Sequential Multiple Assignment Randomized Trial (SMART) study of medication and CBT sequencing in the treatment of pediatric anxiety disorders. BMC Psychiatry.

[124] Barlow D.H., Farchione T.J., Bullis J.R., Gallagher M.W., Murray-Latin H., Sauer-Zavala S., Bentley K.H., Thompson-Hollands J., Conklin L.R., Boswell J.F. (2017). The Unified Protocol for Transdiagnostic Treatment of Emotional Disorders Compared with Diagnosis-Specific Protocols for Anxiety Disorders: A Randomized Clinical Trial. JAMA Psychiatry.

[125] Argyriou E., Prestigiacomo C., Samuel D.B., Stewart J.C., Wu W., Cyders M.A. (2025). Hierarchical taxonomy of psychopathology and personalized mental health treatment selection. Front. Psychiatry.

[126] Myin-Germeys I., Schick A., Ganslandt T., Hajdúk M., Heretik A., Van Hoyweghen I., Kiekens G., Koppe G., Marelli L., Nagyova I. (2024). The experience sampling methodology as a digital clinical tool for more person-centered mental health care: An implementation research agenda. Psychol. Med..

[127] von Klipstein L., Servaas M.N., Schoevers R.A., van der Veen D.C., Riese H. (2023). Integrating personalized experience sampling in psychotherapy: A case illustration of the Therap-i module. Heliyon.

