# Supplementary material for: Mechanistic Non-Response After Psychotherapy for Anxiety Disorders: A Maintenance-Mechanism-Based Clinical Taxonomy
Source: J Clin Med. 2026 May 29;15(11):4223. doi: 10.3390/jcm15114223 (PMC13257802; doi:10.3390/jcm15114223)
Supplement: Supplementary file 1 [file jcm-15-04223-s001.zip › jcm-4337986-supplementary.pdf]

Mechanistic Non-Response After Psychotherapy for Anxiety Disorders: A Maintenance-mechanism-based Clinical Taxonomy

Supplementary Materials file

Supplementary Table S1. SANRA self-check for the structured narrative review

SANRA item	Quality criterion	How the manuscript addresses the criterion	Relevant manuscript component	Self-check status
1	Justification of the article's importance for the readership	The manuscript establishes the clinical importance of anxiety disorders by emphasizing prevalence, early onset, chronicity, functional impairment, comorbidity, and quality-of-life burden. It then identifies a clinically common but insufficiently explained problem: persistent symptoms, relapse, or dropout after evidence-based psychotherapy.	Introduction; epidemiological and burden framing; discussion of non-response rates after CBT; rationale for a clinical taxonomy of mechanistic non-response.	Addressed. The rationale is clinically explicit and directly relevant to psychiatric and psychotherapy readerships.
2	Statement of concrete aims or formulation of questions	The review question is stated explicitly: when apparently adequate evidence-based psychotherapy for an adult anxiety disorder does not produce expected improvement, which pathogenic processes and disrupted therapeutic mechanisms may explain non-response? The aim is not to estimate a pooled treatment effect, but to develop a clinically usable taxonomy of mechanistic non-response.	Methods, review question; Introduction; Table 1 contrasting the proposed framework with adjacent models.	Addressed. The aim is specific, clinically framed, and narrower than a general review of anxiety psychotherapy.
3	Description of the literature search	The Methods section specifies the review design, databases searched, search period, inclusion of landmark pre-2000 papers, search domains, representative PubMed search string, database-specific adaptation, eligibility criteria, study selection, data extraction, evidence mapping, and methodological boundaries. The complete strategy is	Methods sections 2.1 to 2.8; Supplementary Table S1.	Addressed. The search is transparent for a structured narrative review and does not overclaim systematic-review exhaustiveness.

		documented separately in Supplementary Table S1.		
4	Referencing	The manuscript supports key clinical and mechanistic statements with references to diagnostic manuals, clinical guidelines, meta-analyses, systematic reviews, randomized trials, experimental fear-learning literature, neuroimaging and psychophysiological studies, and psychotherapy process research. Foundational papers are used where they define core concepts such as emotional processing, inhibitory learning, avoidance, intolerance of uncertainty, and attachment-related mechanisms.	References; evidence base in sections on psychotherapy mechanisms, pathogenesis, failure modes, biomarkers/predictors, and limitations.	Addressed. Major claims are anchored in peer-reviewed literature, with explicit caution where evidence is indirect or emerging.
5	Scientific reasoning	The manuscript links pathogenic processes to disrupted therapeutic mechanisms through a consistent clinical logic: non-response is examined as a possible failure of inhibitory learning, cognitive flexibility, stress-dependent learning, attentional flexibility, relational engagement, or avoidance reversal. The taxonomy is presented as a formulation aid, not as a validated diagnostic classification or treatment-selection algorithm.	Table 2; Table 4; Figure 1; sections on mechanistic failure modes, treatment matching, limitations, and future directions.	Addressed. The reasoning is internally coherent, mechanism-based, clinically testable, and appropriately cautious.
6	Appropriate presentation of data	The review uses structured tables and a conceptual figure to present evidence and clinical implications: Table 1 positions the framework relative to adjacent models; Table 2 summarizes the six failure modes; Table 4 illustrates disorder-specific expressions; Figure 1 depicts the pathogenesis-to-mechanism-to-outcome model. Evidence strength is discussed qualitatively rather than pooled quantitatively, consistent with the narrative design.	Table 1; Table 2; Table 5; Figure 1; Methods sections on evidence prioritization and narrative synthesis.	Addressed. Evidence is organized in a clinically interpretable format, with limits of inference stated explicitly.

Abbreviations: CBT, cognitive behavioral therapy; SANRA, Scale for the Assessment of Narrative Review Articles.

Supplementary Table S2. Search strategy and evidence mapping

Database	Search string	Mechanism domain
PubMed/MEDLINE	("anxiety disorder*" OR "generalized anxiety disorder" OR "panic disorder" OR "agoraphobia" OR "social anxiety disorder" OR "specific phobia" OR "separation anxiety") AND ("psychotherap*" OR "cognitive behavioral therapy" OR "CBT" OR "exposure therapy" OR "acceptance and commitment therapy" OR "mindfulness" OR "psychodynamic") AND ("treatment response" OR "nonresponse" OR "non-response" OR "remission" OR "relapse" OR "dropout" OR "treatment resistance" OR "treatment failure") AND ("fear extinction" OR "inhibitory learning" OR "expectancy violation" OR "safety behavior*" OR "avoidance")	Fear extinction, inhibitory learning, safety behaviors, avoidance
PubMed/MEDLINE	("anxiety disorder*" OR "generalized anxiety disorder" OR "panic disorder" OR "agoraphobia" OR "social anxiety disorder" OR "specific phobia" OR "separation anxiety") AND ("psychotherap*" OR "cognitive behavioral therapy" OR "CBT" OR "exposure therapy" OR "mindfulness" OR "psychodynamic") AND ("treatment response" OR "nonresponse" OR "remission" OR "relapse" OR "treatment failure") AND ("intolerance of uncertainty" OR "worry" OR "cognitive rigidity" OR "cognitive reappraisal" OR "threat appraisal" OR "probability estimation" OR "reassurance")	Cognitive rigidity, intolerance of uncertainty, threat reappraisal
PubMed/MEDLINE	("anxiety disorder*" OR "generalized anxiety disorder" OR "panic disorder" OR "agoraphobia" OR "social anxiety disorder" OR "specific phobia" OR "separation anxiety") AND ("psychotherap*" OR "CBT" OR "exposure therapy" OR "cognitive behavioral therapy") AND ("treatment response" OR "nonresponse" OR "remission" OR "relapse" OR "dropout") AND ("attentional bias" OR "self-focused attention" OR "interoception" OR "interoceptive monitoring" OR "threat monitoring" OR "attentional control")	Attentional dysregulation, interoceptive monitoring, self-focused attention
PubMed/MEDLINE	("anxiety disorder*" OR "generalized anxiety disorder" OR "panic disorder" OR "agoraphobia" OR "social anxiety disorder" OR "specific phobia") AND ("psychotherap*" OR "CBT" OR "exposure therapy") AND ("treatment response" OR "nonresponse" OR "remission" OR "relapse" OR "dropout") AND ("stress" OR "acute stress" OR "chronic stress" OR "HPA axis" OR "cortisol" OR "hyperarousal" OR "sleep" OR "insomnia" OR "extinction retrieval")	Stress-related learning impairment, HPA axis, sleep, extinction retrieval
PubMed/MEDLINE	("anxiety disorder*" OR "generalized anxiety disorder" OR "panic disorder" OR "agoraphobia" OR "social anxiety disorder" OR "separation anxiety") AND ("psychotherap*" OR "CBT" OR "exposure therapy" OR "psychodynamic" OR "interpersonal") AND ("treatment response" OR "nonresponse" OR "dropout" OR "relapse" OR "treatment failure") AND ("attachment" OR "attachment insecurity" OR "mentalization" OR "therapeutic alliance" OR "rupture" OR "shame" OR "trauma" OR "early adversity")	Attachment-related barriers, shame, alliance rupture, interpersonal learning
PubMed/MEDLINE	("anxiety disorder*" OR "generalized anxiety disorder" OR "panic disorder" OR "agoraphobia" OR "social anxiety disorder" OR "specific phobia") AND ("psychotherap*" OR "CBT" OR "exposure therapy") AND ("treatment response" OR "nonresponse" OR "remission" OR "relapse" OR "dropout") AND ("avoidance" OR "experiential avoidance" OR "safety behavior*" OR "checking" OR "reassurance" OR "accommodation" OR "family accommodation" OR "negative reinforcement")	Chronic avoidance dominance, reinforcement, family accommodation

PubMed/MEDLINE	("anxiety disorder*" OR "generalized anxiety disorder" OR "panic disorder" OR "agoraphobia" OR "social anxiety disorder" OR "specific phobia") AND ("psychotherap*" OR "CBT" OR "exposure therapy") AND ("treatment response" OR "nonresponse" OR "remission" OR "relapse" OR "dropout") AND ("biomarker*" OR "moderator*" OR "mediator*" OR "predictor*" OR "neuroimaging" OR "amygdala" OR "insula" OR "prefrontal cortex" OR "precision psychiatry" OR "precision psychotherapy")	Predictors, moderators, mediators, biomarkers, precision psychotherapy
Scopus	TITLE-ABS-KEY ("anxiety disorder*" OR "generalized anxiety disorder" OR "panic disorder" OR "agoraphobia" OR "social anxiety disorder" OR "specific phobia" OR "separation anxiety") AND TITLE-ABS-KEY ("psychotherap*" OR "cognitive behavioral therapy" OR "CBT" OR "exposure therapy" OR "acceptance and commitment therapy" OR "mindfulness" OR "psychodynamic") AND TITLE-ABS-KEY ("treatment response" OR "nonresponse" OR "non-response" OR "remission" OR "relapse" OR "dropout" OR "treatment resistance" OR "treatment failure") AND TITLE-ABS-KEY ("fear extinction" OR "inhibitory learning" OR "expectancy violation" OR "safety behavior*" OR "avoidance")	Fear extinction, inhibitory learning, safety behaviors, avoidance
Scopus	TITLE-ABS-KEY ("anxiety disorder*" OR "generalized anxiety disorder" OR "panic disorder" OR "agoraphobia" OR "social anxiety disorder" OR "specific phobia" OR "separation anxiety") AND TITLE-ABS-KEY ("psychotherap*" OR "cognitive behavioral therapy" OR "CBT" OR "exposure therapy" OR "mindfulness" OR "psychodynamic") AND TITLE-ABS-KEY ("treatment response" OR "nonresponse" OR "remission" OR "relapse" OR "treatment failure") AND TITLE-ABS-KEY ("intolerance of uncertainty" OR "worry" OR "cognitive rigidity" OR "cognitive reappraisal" OR "threat appraisal" OR "probability estimation" OR "reassurance")	Cognitive rigidity, intolerance of uncertainty, threat reappraisal
Scopus	TITLE-ABS-KEY ("anxiety disorder*" OR "generalized anxiety disorder" OR "panic disorder" OR "agoraphobia" OR "social anxiety disorder" OR "specific phobia" OR "separation anxiety") AND TITLE-ABS-KEY ("psychotherap*" OR "CBT" OR "exposure therapy" OR "cognitive behavioral therapy") AND TITLE-ABS-KEY ("treatment response" OR "nonresponse" OR "remission" OR "relapse" OR "dropout") AND TITLE-ABS-KEY ("attentional bias" OR "self-focused attention" OR "interoception" OR "interoceptive monitoring" OR "threat monitoring" OR "attentional control")	Attentional dysregulation, interoceptive monitoring, self-focused attention
Scopus	TITLE-ABS-KEY ("anxiety disorder*" OR "generalized anxiety disorder" OR "panic disorder" OR "agoraphobia" OR "social anxiety disorder" OR "specific phobia") AND TITLE-ABS-KEY ("psychotherap*" OR "CBT" OR "exposure therapy") AND TITLE-ABS-KEY ("treatment response" OR "nonresponse" OR "remission" OR "relapse" OR "dropout") AND TITLE-ABS-KEY ("stress" OR "acute stress" OR "chronic stress" OR "HPA axis" OR "cortisol" OR "hyperarousal" OR "sleep" OR "insomnia" OR "extinction retrieval")	Stress-related learning impairment, HPA axis, sleep, extinction retrieval
Scopus	TITLE-ABS-KEY ("anxiety disorder*" OR "generalized anxiety disorder" OR "panic disorder" OR "agoraphobia" OR "social anxiety disorder" OR "separation anxiety") AND TITLE-ABS-KEY ("psychotherap*" OR "CBT" OR "exposure therapy" OR "psychodynamic" OR "interpersonal") AND TITLE-ABS-KEY ("treatment response" OR "nonresponse" OR "dropout" OR "relapse" OR "treatment failure") AND TITLE-ABS-KEY ("attachment" OR "attachment insecurity" OR "mentalization" OR "therapeutic	Attachment-related barriers, shame, alliance rupture, interpersonal learning

	alliance" OR "rupture" OR "shame" OR "trauma" OR "early adversity")	
Scopus	TITLE-ABS-KEY ("anxiety disorder*" OR "generalized anxiety disorder" OR "panic disorder" OR "agoraphobia" OR "social anxiety disorder" OR "specific phobia") AND TITLE-ABS-KEY ("psychotherap*" OR "CBT" OR "exposure therapy") AND TITLE-ABS-KEY ("treatment response" OR "nonresponse" OR "remission" OR "relapse" OR "dropout") AND TITLE-ABS-KEY ("avoidance" OR "experiential avoidance" OR "safety behavior*" OR "checking" OR "reassurance" OR "accommodation" OR "family accommodation" OR "negative reinforcement")	Chronic avoidance dominance, reinforcement, family accommodation
Scopus	TITLE-ABS-KEY ("anxiety disorder*" OR "generalized anxiety disorder" OR "panic disorder" OR "agoraphobia" OR "social anxiety disorder" OR "specific phobia") AND TITLE-ABS-KEY ("psychotherap*" OR "CBT" OR "exposure therapy") AND TITLE-ABS-KEY ("treatment response" OR "nonresponse" OR "remission" OR "relapse" OR "dropout") AND TITLE-ABS-KEY ("biomarker*" OR "moderator*" OR "mediator*" OR "predictor*" OR "neuroimaging" OR "amygdala" OR "insula" OR "prefrontal cortex" OR "precision psychiatry" OR "precision psychotherapy")	Predictors, moderators, mediators, biomarkers, precision psychotherapy
PsycINFO	TI,AB,SU ("anxiety disorder*" OR "generalized anxiety disorder" OR "panic disorder" OR "agoraphobia" OR "social anxiety disorder" OR "specific phobia" OR "separation anxiety") AND TI,AB,SU ("psychotherap*" OR "cognitive behavioral therapy" OR "CBT" OR "exposure therapy" OR "acceptance and commitment therapy" OR "mindfulness" OR "psychodynamic") AND TI,AB,SU ("treatment response" OR "nonresponse" OR "non-response" OR "remission" OR "relapse" OR "dropout" OR "treatment resistance" OR "treatment failure") AND TI,AB,SU ("fear extinction" OR "inhibitory learning" OR "expectancy violation" OR "safety behavior*" OR "avoidance")	Fear extinction, inhibitory learning, safety behaviors, avoidance
PsycINFO	TI,AB,SU ("anxiety disorder*" OR "generalized anxiety disorder" OR "panic disorder" OR "agoraphobia" OR "social anxiety disorder" OR "specific phobia" OR "separation anxiety") AND TI,AB,SU ("psychotherap*" OR "cognitive behavioral therapy" OR "CBT" OR "exposure therapy" OR "mindfulness" OR "psychodynamic") AND TI,AB,SU ("treatment response" OR "nonresponse" OR "remission" OR "relapse" OR "treatment failure") AND TI,AB,SU ("intolerance of uncertainty" OR "worry" OR "cognitive rigidity" OR "cognitive reappraisal" OR "threat appraisal" OR "probability estimation" OR "reassurance")	Cognitive rigidity, intolerance of uncertainty, threat reappraisal
PsycINFO	TI,AB,SU ("anxiety disorder*" OR "generalized anxiety disorder" OR "panic disorder" OR "agoraphobia" OR "social anxiety disorder" OR "specific phobia" OR "separation anxiety") AND TI,AB,SU ("psychotherap*" OR "CBT" OR "exposure therapy" OR "cognitive behavioral therapy") AND TI,AB,SU ("treatment response" OR "nonresponse" OR "remission" OR "relapse" OR "dropout") AND TI,AB,SU ("attentional bias" OR "self-focused attention" OR "interoception" OR "interoceptive monitoring" OR "threat monitoring" OR "attentional control")	Attentional dysregulation, interoceptive monitoring, self-focused attention
PsycINFO	TI,AB,SU ("anxiety disorder*" OR "generalized anxiety disorder" OR "panic disorder" OR "agoraphobia" OR "social anxiety disorder" OR "specific phobia") AND TI,AB,SU ("psychotherap*" OR "CBT" OR "exposure therapy") AND TI,AB,SU ("treatment response" OR "nonresponse" OR "remission" OR "relapse" OR "dropout") AND TI,AB,SU ("stress" OR "acute stress" OR "chronic stress" OR "HPA	Stress-related learning impairment, HPA axis, sleep, extinction retrieval

	axis" OR "cortisol" OR "hyperarousal" OR "sleep" OR "insomnia" OR "extinction retrieval")	
PsycINFO	TI,AB,SU ("anxiety disorder*" OR "generalized anxiety disorder" OR "panic disorder" OR "agoraphobia" OR "social anxiety disorder" OR "separation anxiety") AND TI,AB,SU ("psychotherap*" OR "CBT" OR "exposure therapy" OR "psychodynamic" OR "interpersonal") AND TI,AB,SU ("treatment response" OR "nonresponse" OR "dropout" OR "relapse" OR "treatment failure") AND TI,AB,SU ("attachment" OR "attachment insecurity" OR "mentalization" OR "therapeutic alliance" OR "rupture" OR "shame" OR "trauma" OR "early adversity")	Attachment-related barriers, shame, alliance rupture, interpersonal learning
PsycINFO	TI,AB,SU ("anxiety disorder*" OR "generalized anxiety disorder" OR "panic disorder" OR "agoraphobia" OR "social anxiety disorder" OR "specific phobia") AND TI,AB,SU ("psychotherap*" OR "CBT" OR "exposure therapy") AND TI,AB,SU ("treatment response" OR "nonresponse" OR "remission" OR "relapse" OR "dropout") AND TI,AB,SU ("avoidance" OR "experiential avoidance" OR "safety behavior*" OR "checking" OR "reassurance" OR "accommodation" OR "family accommodation" OR "negative reinforcement")	Chronic avoidance dominance, reinforcement, family accommodation
PsycINFO	TI,AB,SU ("anxiety disorder*" OR "generalized anxiety disorder" OR "panic disorder" OR "agoraphobia" OR "social anxiety disorder" OR "specific phobia") AND TI,AB,SU ("psychotherap*" OR "CBT" OR "exposure therapy") AND TI,AB,SU ("treatment response" OR "nonresponse" OR "remission" OR "relapse" OR "dropout") AND TI,AB,SU ("biomarker*" OR "moderator*" OR "mediator*" OR "predictor*" OR "neuroimaging" OR "amygdala" OR "insula" OR "prefrontal cortex" OR "precision psychiatry" OR "precision psychotherapy")	Predictors, moderators, mediators, biomarkers, precision psychotherapy
Web of Science	TS=("anxiety disorder*" OR "generalized anxiety disorder" OR "panic disorder" OR "agoraphobia" OR "social anxiety disorder" OR "specific phobia" OR "separation anxiety") AND TS=("psychotherap*" OR "cognitive behavioral therapy" OR "CBT" OR "exposure therapy" OR "acceptance and commitment therapy" OR "mindfulness" OR "psychodynamic") AND TS=("treatment response" OR "nonresponse" OR "non-response" OR "remission" OR "relapse" OR "dropout" OR "treatment resistance" OR "treatment failure") AND TS=("fear extinction" OR "inhibitory learning" OR "expectancy violation" OR "safety behavior*" OR "avoidance")	Fear extinction, inhibitory learning, safety behaviors, avoidance
Web of Science	TS=("anxiety disorder*" OR "generalized anxiety disorder" OR "panic disorder" OR "agoraphobia" OR "social anxiety disorder" OR "specific phobia" OR "separation anxiety") AND TS=("psychotherap*" OR "cognitive behavioral therapy" OR "CBT" OR "exposure therapy" OR "mindfulness" OR "psychodynamic") AND TS=("treatment response" OR "nonresponse" OR "remission" OR "relapse" OR "treatment failure") AND TS=("intolerance of uncertainty" OR "worry" OR "cognitive rigidity" OR "cognitive reappraisal" OR "threat appraisal" OR "probability estimation" OR "reassurance")	Cognitive rigidity, intolerance of uncertainty, threat reappraisal
Web of Science	TS=("anxiety disorder*" OR "generalized anxiety disorder" OR "panic disorder" OR "agoraphobia" OR "social anxiety disorder" OR "specific phobia" OR "separation anxiety") AND TS=("psychotherap*" OR "CBT" OR "exposure therapy" OR "cognitive behavioral therapy") AND TS=("treatment response" OR "nonresponse" OR "remission" OR "relapse" OR "dropout") AND TS=("attentional bias" OR "self-focused attention" OR	Attentional dysregulation, interoceptive monitoring, self-focused attention

	"interoception" OR "interoceptive monitoring" OR "threat monitoring" OR "attentional control")	
Web of Science	TS=("anxiety disorder*" OR "generalized anxiety disorder" OR "panic disorder" OR "agoraphobia" OR "social anxiety disorder" OR "specific phobia") AND TS=("psychotherap*" OR "CBT" OR "exposure therapy") AND TS=("treatment response" OR "nonresponse" OR "remission" OR "relapse" OR "dropout") AND TS=("stress" OR "acute stress" OR "chronic stress" OR "HPA axis" OR "cortisol" OR "hyperarousal" OR "sleep" OR "insomnia" OR "extinction retrieval")	Stress-related learning impairment, HPA axis, sleep, extinction retrieval
Web of Science	TS=("anxiety disorder*" OR "generalized anxiety disorder" OR "panic disorder" OR "agoraphobia" OR "social anxiety disorder" OR "separation anxiety") AND TS=("psychotherap*" OR "CBT" OR "exposure therapy" OR "psychodynamic" OR "interpersonal") AND TS=("treatment response" OR "nonresponse" OR "dropout" OR "relapse" OR "treatment failure") AND TS=("attachment" OR "attachment insecurity" OR "mentalization" OR "therapeutic alliance" OR "rupture" OR "shame" OR "trauma" OR "early adversity")	Attachment-related barriers, shame, alliance rupture, interpersonal learning
Web of Science	TS=("anxiety disorder*" OR "generalized anxiety disorder" OR "panic disorder" OR "agoraphobia" OR "social anxiety disorder" OR "specific phobia") AND TS=("psychotherap*" OR "CBT" OR "exposure therapy") AND TS=("treatment response" OR "nonresponse" OR "remission" OR "relapse" OR "dropout") AND TS=("avoidance" OR "experiential avoidance" OR "safety behavior*" OR "checking" OR "reassurance" OR "accommodation" OR "family accommodation" OR "negative reinforcement")	Chronic avoidance dominance, reinforcement, family accommodation
Web of Science	TS=("anxiety disorder*" OR "generalized anxiety disorder" OR "panic disorder" OR "agoraphobia" OR "social anxiety disorder" OR "specific phobia") AND TS=("psychotherap*" OR "CBT" OR "exposure therapy") AND TS=("treatment response" OR "nonresponse" OR "remission" OR "relapse" OR "dropout") AND TS=("biomarker*" OR "moderator*" OR "mediator*" OR "predictor*" OR "neuroimaging" OR "amygdala" OR "insula" OR "prefrontal cortex" OR "precision psychiatry" OR "precision psychotherapy")	Predictors, moderators, mediators, biomarkers, precision psychotherapy
Cochrane Library	("anxiety disorder*" OR "generalized anxiety disorder" OR "panic disorder" OR "agoraphobia" OR "social anxiety disorder" OR "specific phobia" OR "separation anxiety") AND ("psychotherapy" OR "cognitive behavioral therapy" OR "CBT" OR "exposure therapy") AND ("treatment response" OR "nonresponse" OR "remission" OR "relapse" OR "dropout" OR "treatment failure") AND ("fear extinction" OR "inhibitory learning" OR "safety behavior*" OR "avoidance")	Fear extinction, inhibitory learning, safety behaviors, avoidance
Cochrane Library	("anxiety disorder*" OR "generalized anxiety disorder" OR "panic disorder" OR "agoraphobia" OR "social anxiety disorder" OR "specific phobia") AND ("psychotherapy" OR "cognitive behavioral therapy" OR "CBT" OR "exposure therapy") AND ("treatment response" OR "nonresponse" OR "remission" OR "relapse" OR "treatment failure") AND ("intolerance of uncertainty" OR "worry" OR "cognitive reappraisal" OR "threat appraisal" OR "reassurance")	Cognitive rigidity, intolerance of uncertainty, threat reappraisal
Cochrane Library	("anxiety disorder*" OR "generalized anxiety disorder" OR "panic disorder" OR "agoraphobia" OR "social anxiety disorder" OR "specific phobia") AND ("psychotherapy" OR "cognitive behavioral therapy" OR "CBT" OR "exposure therapy") AND ("treatment	Attentional dysregulation, interoceptive monitoring, self-

	response" OR "nonresponse" OR "relapse" OR "dropout") AND ("attentional bias" OR "self-focused attention" OR "interoception" OR "interoceptive monitoring" OR "attentional control")	focused attention
Cochrane Library	("anxiety disorder*" OR "generalized anxiety disorder" OR "panic disorder" OR "agoraphobia" OR "social anxiety disorder" OR "specific phobia") AND ("psychotherapy" OR "cognitive behavioral therapy" OR "CBT" OR "exposure therapy") AND ("treatment response" OR "nonresponse" OR "relapse" OR "dropout") AND ("stress" OR "HPA axis" OR "cortisol" OR "hyperarousal" OR "sleep" OR "insomnia" OR "extinction retrieval")	Stress-related learning impairment, HPA axis, sleep, extinction retrieval
Cochrane Library	("anxiety disorder*" OR "generalized anxiety disorder" OR "panic disorder" OR "agoraphobia" OR "social anxiety disorder" OR "separation anxiety") AND ("psychotherapy" OR "CBT" OR "exposure therapy" OR "psychodynamic" OR "interpersonal") AND ("treatment response" OR "nonresponse" OR "dropout" OR "relapse" OR "treatment failure") AND ("attachment" OR "mentalization" OR "therapeutic alliance" OR "rupture" OR "shame" OR "trauma" OR "early adversity")	Attachment-related barriers, shame, alliance rupture, interpersonal learning
Cochrane Library	("anxiety disorder*" OR "generalized anxiety disorder" OR "panic disorder" OR "agoraphobia" OR "social anxiety disorder" OR "specific phobia") AND ("psychotherapy" OR "CBT" OR "exposure therapy") AND ("treatment response" OR "nonresponse" OR "remission" OR "relapse" OR "dropout") AND ("avoidance" OR "experiential avoidance" OR "safety behavior*" OR "checking" OR "reassurance" OR "accommodation" OR "family accommodation")	Chronic avoidance dominance, reinforcement, family accommodation
Cochrane Library	("anxiety disorder*" OR "generalized anxiety disorder" OR "panic disorder" OR "agoraphobia" OR "social anxiety disorder" OR "specific phobia") AND ("psychotherapy" OR "CBT" OR "exposure therapy") AND ("treatment response" OR "nonresponse" OR "remission" OR "relapse" OR "dropout") AND ("biomarker*" OR "moderator*" OR "mediator*" OR "predictor*" OR "neuroimaging" OR "amygdala" OR "insula" OR "prefrontal cortex" OR "precision psychiatry" OR "precision psychotherapy")	Predictors, moderators, mediators, biomarkers, precision psychotherapy
Backward citation searching	Reference lists of included systematic reviews, meta-analyses, randomized trials, major theoretical papers, and clinical guidelines were hand-searched for earlier landmark studies and highly relevant mechanistic papers not retrieved by database searches.	Foundational models, landmark studies, clinical guidelines
Forward citation checking	Key conceptual and mechanistic papers on emotional processing theory, inhibitory learning, intolerance of uncertainty, avoidance, attentional bias, attachment, and psychotherapy response prediction were checked for subsequent highly relevant citations.	Citation expansion and evidence triangulation

Abbreviations: CBT, cognitive behavioral therapy; HPA, hypothalamic-pituitary-adrenal; SANRA, Scale for the Assessment of Narrative Review Articles.

Supplementary Table S3. Clinician Rapid Guide: Mechanistic Non-Response After Psychotherapy for Anxiety Disorders

Failure signature after apparently adequate psychotherapy	Likely failure mode	First clinical question	First-line adaptation
Exposure was completed, but feared beliefs did not change; gains remain context-bound or fear rapidly returns	Impaired inhibitory learning	Was the feared expectancy actually tested, and were safety behaviors still active?	Redesign exposure around expectancy violation, remove safety behaviors, vary contexts, and strengthen retrieval/generalization
Sessions become repetitive debates; reassurance helps briefly; patient cannot act until doubt is eliminated	Cognitive rigidity / intolerance of uncertainty	Is the problem distorted probability, or inability to tolerate unresolved possibility?	Shift from probability disputation to uncertainty exposure, behavioral experiments, metacognitive work, and reassurance reduction
Treatment evokes flooding, shutdown, dissociation, poor recall, or worsening sleep	Stress-related learning impairment	Are arousal, sleep, stress load, or sedative effects preventing encoding and consolidation?	Stabilize sleep/arousal, review medication/sedative timing, titrate exposure, and sequence learning tasks carefully
Patient performs tasks but remains internally focused, threat-scanning, or unable to recall corrective cues	Attentional dysregulation	Where was attention during the therapeutic task?	Add external-focus training, attentional flexibility work, interoceptive attention practice, and video feedback when appropriate
Superficial compliance, limited disclosure, shame, mistrust, rupture sensitivity, or withdrawal after perceived criticism	Attachment-related barriers	Is the therapeutic relationship itself activating threat?	Prioritize alliance repair, validation before challenge, relational formulation, and work with shame/mistrust
Homework repeatedly fails despite agreement; life remains organized around avoidance and accommodation	Chronic avoidance dominance	What does avoidance accomplish, and who or what reinforces it?	Use functional analysis, values-based activation, contingency planning, reduction of accommodation, and graded exposure embedded in meaningful goals

Footnote: These prompts are intended for clinical formulation and monitoring. They should not be used as validated diagnostic categories or as an automated treatment-selection algorithm.
